# CaMKII activation participates in doxorubicin cardiotoxicity and is attenuated by moderate GRP78 overexpression

**DOI:** 10.1371/journal.pone.0215992

**Published:** 2019-04-29

**Authors:** Henrike Tscheschner, Eric Meinhardt, Philipp Schlegel, Andreas Jungmann, Lorenz H. Lehmann, Oliver J. Müller, Patrick Most, Hugo A. Katus, Philip W. Raake

**Affiliations:** 1 Department of Internal Medicine III, Cardiology, University Hospital Heidelberg, University of Heidelberg, Heidelberg, Germany; 2 DZHK (German Centre for Cardiovascular Research), Partner Site Heidelberg/Mannheim, Heidelberg, Germany; 3 Department of Internal Medicine III, Cardiology, Angiology and Intensive Care Medicine, University Hospital Kiel, Kiel, Germany; University of PECS Medical School, HUNGARY

## Abstract

The clinical use of the chemotherapeutic doxorubicin (Dox) is limited by cardiotoxic side-effects. One of the early Dox effects is induction of a sarcoplasmic reticulum (SR) Ca^2+^ leak. The chaperone Glucose regulated protein 78 (GRP78) is important for Ca^2+^ homeostasis in the endoplasmic reticulum (ER)—the organelle corresponding to the SR in non-cardiomyocytes—and has been shown to convey resistance to Dox in certain tumors. Our aim was to investigate the effect of cardiac GRP78 gene transfer on Ca^2+^ dependent signaling, cell death, cardiac function and survival in clinically relevant *in vitro* and *in vivo* models for Dox cardiotoxicity.By using neonatal cardiomyocytes we could demonstrate that Dox induced Ca^2+^ dependent Ca^2+^ /calmodulin-dependent protein kinase II (CaMKII) activation is one of the factors involved in Dox cardiotoxicity by promoting apoptosis. Furthermore, we found that adeno-associated virus (AAV) mediated GRP78 overexpression partly protects neonatal cardiomyocytes from Dox induced cell death by modulating Ca^2+^ dependent pathways like the activation of CaMKII, phospholamban (PLN) and p53 accumulation. Most importantly, cardiac GRP78 gene therapy in mice treated with Dox revealed improved diastolic function (dP/dtmin) and survival after Dox treatment. In conclusion, our results demonstrate for the first time that Ca^2+^ dependent CaMKII activation fosters Dox cardiomyopathy and provide additional insight into possible mechanisms by which GRP78 overexpression protects cardiomyocytes from Doxorubicin toxicity.

## Introduction

The anthracycline doxorubicin (Dox) is an effective and thus frequently applied anticancer treatment. However, Dox treatment comes with severe adverse effects substantially limiting its use as chemotherapeutic. The risk of developing cardiomyopathy increases with the cumulative dose [1]. Molecular mechanisms are still controversial and therapeutic options are still limited and mainly restricted to symptomatic approaches.

The underlying cause for Dox cardiomyopathy is thought to be the loss of cardiomyocytes due to apoptosis. One of the early events in Dox cardiotoxicity is induction of a diastolic Ca^2+^ leak from the sarcoplasmic reticulum (SR) with elevated diastolic Ca^2+^ levels in the cytosol and depletion of SR Ca^2+^ [2, 3]. Furthermore, Ca^2+^ is tightly linked to the activation of Ca^2+^/Calmodulin dependent kinase II (CaMKII), which was recently related to cardiac apoptosis and the development of heart failure [4]. Ca^2+^ dependent signaling including CaMKII activation could thus be a major triggering factor in Dox cardiomyopathy and its repression of potential therapeutic benefit [2, 3].

The chaperone Glucose regulated protein 78 (GRP78), which is a central mediator of the unfolded protein response during endoplasmic reticulum (ER) stress, is a potential candidate for gene therapy in Dox cardiotoxicity for two reasons: First, it confers chemoresistance in certain tumors and tumor associated cell lines, while knockdown of GRP78 on the other hand resensitizes tumor cells to Dox [5, 6]. Second, GRP78 regulates Ca^2+^ homeostasis and flux from the endoplasmic reticulum (ER) to mitochondria via its interaction with the Phospho-inositol-3 Receptor (IP3R)[7]. The SR, which is involved in Dox cardiotoxicity is a specialized type of ER in cardiomyocytes, with enhanced Ca^2+^ storage capacity. In a recent publication it was already shown that Dox treatment impairs the protective ER stress response and that restoration of GRP78 expression reduces ER stress induced cell death after Dox treatment [8].

Therefore, our aim was to further investigate possible mechanisms and pathways involved in the protective effect of GRP78 on Dox cardiotoxicity focusing on Ca^2+^ dependent apoptotic pathways *in vivo*. To this end we examined the effects of targeted adeno-associated virus (AAV) mediated GRP78 overexpression *in vitro* and functionally *in vivo*.

## Materials and methods

### Reagents

If not further specified, chemicals were purchased from SIGMA-Aldrich, Munich, Germany.

### Animal model of doxorubicin cardiotoxicity and gene therapy

The present investigation was carried out according to the Guide for the Care and Use of laboratory Animals and was approved by the Animal Care and Use Committee of the state of Baden-Württemberg (Protocol Nr. G128/09, Regierungspräsidium Karlsruhe) and had been performed in accordance with the ethical standards laid down in the 1964 Declaration of Helsinki and its later amendments, including the Directive 2010/63/EU of the European Parliament. The animals were kept under specific pathogen free environment in the Interfacultary Biomedical Faculty Heidelberg in a 12h sleep-wake cycle. Food and water were provided *ad libitum*. Anesthesia was monitored by checking the corneal reflex and pinching the interdigital skin.

7 weeks old male C57/Bl6JN mice purchased from Janvier Labs (France) were tail-vein injected with 3x10^12^ virus genomes. The GRP78 group received an AAV serotype 9 expressing GRP78 under the control of a CMV-enhanced myosin light chain (CMV-MLC) 1.8kb promoter (AAV-GRP78) and the control group received an AAV expressing Luciferase under control of the same promoter (AAV-Luc) (S1 File). One day before Dox treatment, ejection fraction was measured using echocardiography. 3 weeks after virus treatment, mice were i.p.-injected with Dox (2mg/mL stock solution for animal use) once a week with a cumulative dose of 20mg/kg (week 1: 10mg/kg, weeks 2 and 3: 5mg/kg). Sterile 0.9% NaCl solution (B.Braun, Melsungen, Germany) was used as a control.

The experiment was terminated when 40% of Dox-treated animals had died, typically 2–3 weeks after the last Dox treatment. One day before termination, ejection fraction was measured. The next day, animals were anesthetized (i.p-injection of 500μg/kg body weight Medetomidine, 5mg/kg Midazolam and 50μg/kg Fentanyl) and intraventricular pressure-volume (PV) loops were derived. After PV loop measurement, blood samples were taken, mice were sacrificed by cervical dislocation and the hearts were harvested.

### Measurement of high sensitive Troponin T (hsTnT)

Animals were anesthetized with 5% Isoflurane. 200μL blood was taken from the retrobulbary venous plexus through a heparinized capillary and centrifuged at 2500rpm, 4°C 15min. Plasma was diluted 1:40 with 0.9% NaCl solution (B.Braun) and analyzed at the diagnostic laboratory for clinical chemistry of the University Hospital Heidelberg with a clinical hsTnT assay (Roche, Mannheim, Germany).

### Isolation of neonatal rat ventricular cardiomyocytes (NRVCM)

1–3 days old rats were decapitated using a sharp surgical sissor. NRVCM were isolated from the hearts as described in the supplement (S1 File). Cardiomyocytes were separated from fibroblasts by centrifugation in a Percoll (GE Healthcare Sciences, Freiburg, Germany) gradient and kept in M199 supplemented with fetal calf serum (FCS) (Biochrom/Merck, Berlin, Germany), L-Glutamine (Life Technologies GmbH, Darmstadt, Germany), CaCl_2_ (SIGMA-Aldrich) and Penicillin/Streptomycin (Life Technologies GmbH).

### Doxorubicin treatment

3 or 5 days after plating of NRVCM aliquoted and freshly thawed Dox (Sigma Aldrich, München) was diluted in M199 + 0.5% FCS (S1 File). Concentrations between 0.5μM and 1μM were used.

For experiments with chelation of free Ca^2+^ the Ca^2+^ chelator 1,2-Bis(2-aminophenoxy)ethane-N,N,N′,N′-tetraacetic acid tetrakis—acetoxymethyl ester (BAPTA-AM, Thermo Scientific, Schwert) was used. 20μM BAPTA-AM or Dimethylsulfoxid (DMSO, Sigma Aldrich, München) for untreated controls were added 45min before treatment with Dox. BAPTA-AM was present for the rest of the experiment.

For specific CaMKII inhibition the CaMKII inhibitor myristoylated autocamtide-2-related inhibitory peptide (AIP) (Calbiochem/Merck/Millipore, Darmstadt, Germany) was used at a concentration of 5μM 14h after starting Dox treatment.

### Transduction of NRVCM with AAVs

Cells were transduced with an AAV serotype 6 expressing GRP78 (AAV-GRP78) or Luciferase (AAV-Luc) under control of the CMV-MLC promoter at day 3 after plating. A multiplicity of infection (MOI) ranging from 2.5x10^3^ to 15x10^4^ was used. Medium was renewed 48h after transduction with the addition of Dox.

### ToxiLight assay

The Adenylatkinase-based Kit ToxiLight Non-destructive Cytotoxicity BioAssay (Lonza, Basel, Switzerland) was used to measure cell death. Measurements from cell culture supernatant were done in triplicates in a 384-well plate and according to the manufacturer’s instructions.

### TdT-mediated dUTP-Biotin nick end labeling (TUNEL) assay

TUNEL Assay was performed with 0.6x10^5^ cells seeded on Laminin-coated glass coverslips in a 12-well plate using the In Situ Cell Death Detection Kit TMR red (Roche, Mannheim, Germany) according to the manufacturer’s instructions. 8 fluorescence images were taken and TUNEL-positive nuclei versus total nuclei were analyzed using ImageJ software.

### Caspase 9 and apoptosis inducing factor (AIF) staining in vitro

The assay was performed as described in the supplement (S1 File). In brief, 1.5x10^4^ NRVCM were seeded on a 96-well plate pre-coated with Collagen-A. After treatment, cells were fixed, blocked and incubated with primary antibodies (anti-AIF, anti-Caspase 9 and anti-α actinin). The cells were washed and stained with fluorescent secondary antibodies. DAPI was added to stain nuclei. Fluorescent microscopy images were taken. For the AIF translocation assay, the signal intensity of nuclear and cytosolic AIF was determined using ImageJ and the ratio of nuclear to cytosolic AIF was calculated. For caspase 9 activation assay, the number of caspase-9 positive cells was determined and the ratio between caspase 9-positive cells and total cell number was calculated.

### Reactive oxygen species (ROS) detection

For measurement of ROS from cell culture supernatant, ROS-Glo Assay (Promega, Mannheim, Germany) was used. 4x10^5^ cells/well were used in a 24-well format. 6h before ending the experiment, 30μL H_2_O_2_ substrate was added to the cell culture medium. ROS levels were normalized relative to the cell number determined by Sulfrhodamine assay (S1 File).

### Ca^2+^ transients and SR Ca^2+^ content

100 000 NRVCM mixed with laminin 1:100 were plated in a 10mm well fluoro dish (World Precision Instruments, Berlin, Germany). Dox treatment was done in M199 modified with 0.5% FCS (M199 + 5mM taurine, 5mM carnitin, 5mM creatine, 1% pen/strep (Life Technologies), Insulin 25 I.U./500ml (Insuman Rapid, Sanofi-Aventis, Frankfurt (Main), Germany) and 5mM mercaptopropionylglycin). In the following serum free M199 was used. Cells were loaded with FURA-2A for 15min and stimulated in an electric field with 1Hz. After 12 pulses 340/380 nm ratios were recorded using an Olympus IX70 Fluorescence Microscope (Olympus Deutschland GmbH, Hamburg, Germany). Three sequences with three Ca^2+^ transients were recorded. SR Ca^2+^ content was determined by supplementing the medium with 10mM caffeine. The height of the caffeine induced Ca^2+^ peak was taken as index for SR Ca^2+^ content. Transients and Caffein peaks were analyzed using the Labchart 6 Software (ADInstruments, Oxford, UK).

### Statistics

Statistical analysis was performed using the GraphPad Prism analysis Software (GraphPad Software Inc., La Jolla, USA). The statistical tests used are indicated in the Figure legends. P< 0.05 was regarded as significant. Data are depicted as mean ± SEM.

Additional methods can be found in the online supplement (S1 File).

## Results

### Dox suppresses GRP78 expression in cardiomyocytes

**Fig 1:**
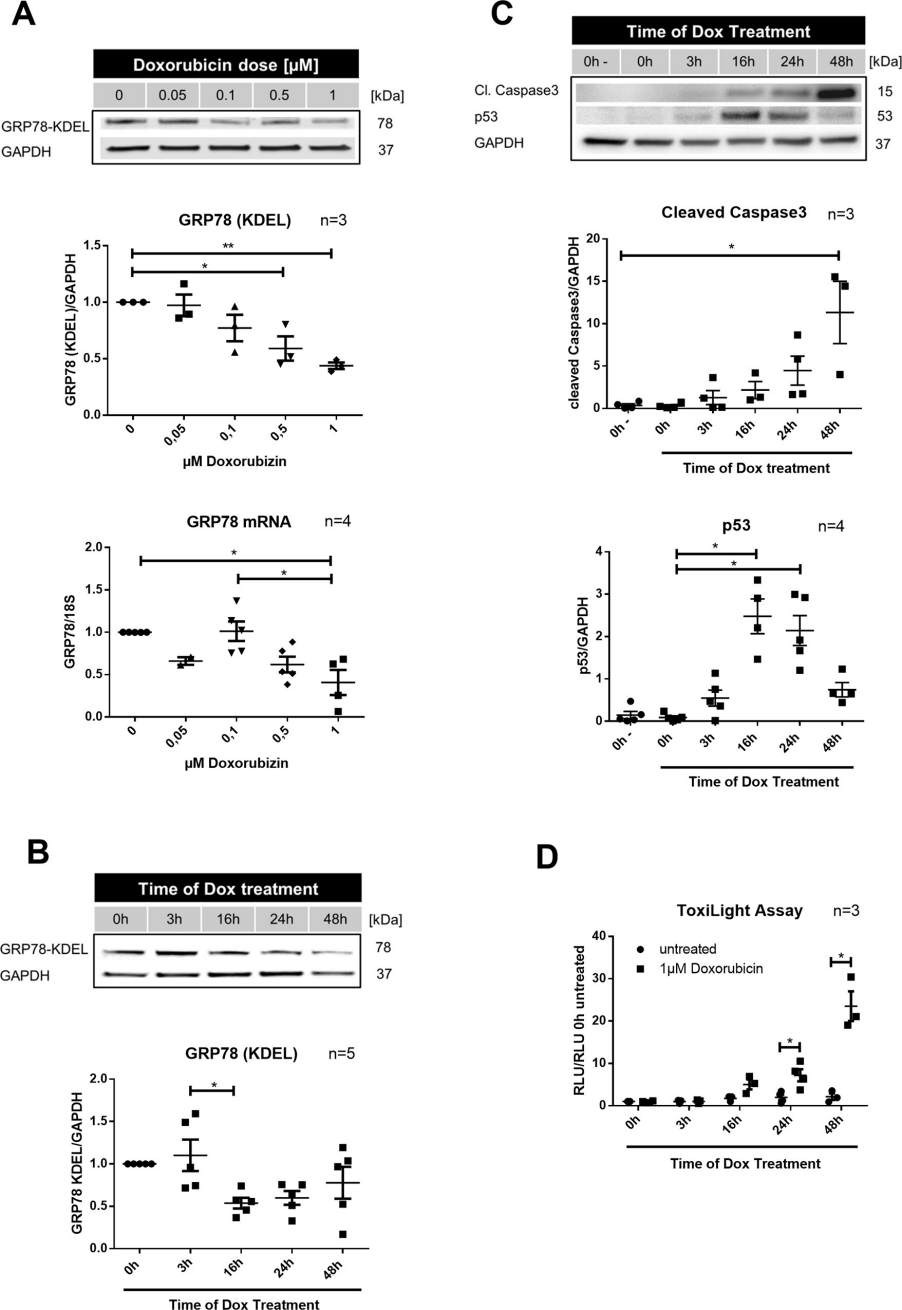
Timeframe of doxorubicin (Dox) induced cell death and apoptosis. (**A**) Neonatal rat cardiomyocytes were treated with indicated doses of Dox for 24h. Glucose regulated protein 78 (GRP78; n = 3) was detected with an KDEL antibody. RNA expression (n = 4) was determined by Realtime-PCR with 18s mRNA as housekeeping gene. Samples were normalized to untreated control. (**B**) Time dependent changes in GRP78 protein expression (n = 5) after treatment with 1**μ**M Dox (detected with KDEL antibody). (**C**) Cleaved caspase3 (n = 3) and p53 levels (n = 4) were detected by Western Blot. Glyceraldehyde 3-phosphate dehydrogenase (GAPDH) was used as housekeeping gene. (**D**) Determination of time dependent cell death via ToxiLight Assay after administration of 1**μ**M Dox (n = 3). Relative light units (RLU) were normalized to RLU of untreated control sample. *p < 0.05 for 2-way ANOVA with Bonferroni post hoc testing. **p < 0.05 for 1-way ANOVA with Bonferroni post hoc testing.

As our aim was to investigate the potential of prophylactic GRP78 overexpression, we were first interested in changes of endogenous GRP78 expression in Dox treated NRVCMs. A clear dose and time dependent downregulation of GRP78 could be observed, with first changes in GRP78 levels occurring after 16h (Fig 1A and 1B). Realtime-PCR confirmed a dose dependent reduction of GRP78 expression by Dox (Fig 1A).

Interestingly, GRP78 downregulation coincided with the occurrence of cell death. Cell death, measured by ToxiLight Assay from the cell culture supernatant, started to increase 16h after initiation of Dox treatment and peaked after 48h (Fig 1D). As a specific sign for apoptotic cell death caspase3 cleavage already appeared after 3h Dox treatment and also peaked at 48h (Fig 1C). In addition, in the Dox treated NRVCM we could detect a strong accumulation of p53, which appeared synchronously with the first caspase3 activation and peaked at 16-24h (Fig 1C bottom).

*In vivo* we saw similar effects on GRP78 expression with Dox treatment (15 mg/kg and 20 mg/kg respectively). After 24h GRP78 mRNA levels started to decrease resulting in a significant downregulation at day 5 after Dox administration. GRP78 protein expression was significantly reduced after 5 days (S3 Fig).

### Dox cardiotoxicity is associated with disturbed SR homeostasis and activation of CaMKII

**Fig 2:**
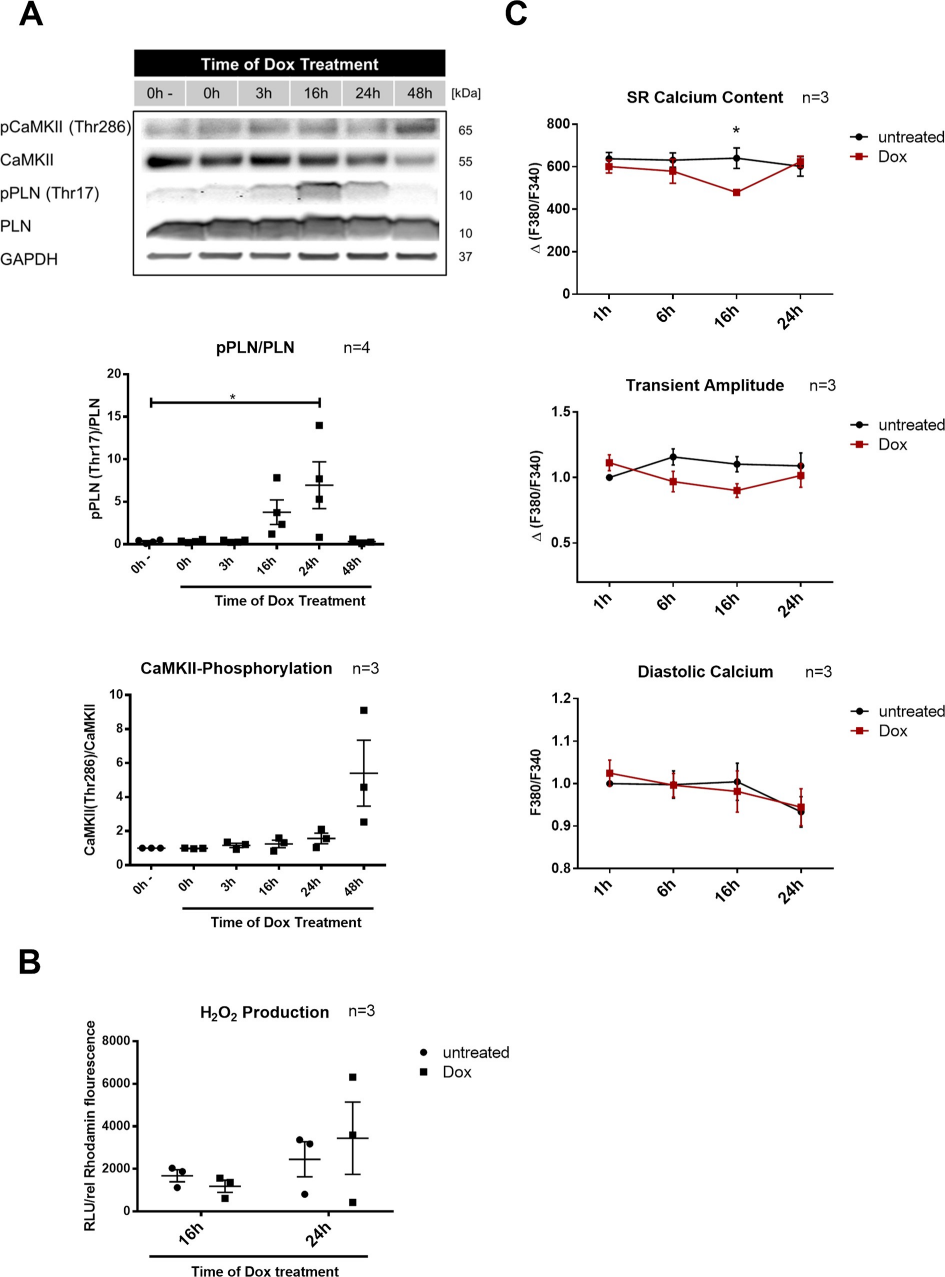
Disturbed Ca^2+^ handling and Ca^2+^/calmodulin-dependent protein kinase II (CaMKII) activation by Doxorubicin (Dox) treatment. Neonatal rat cardiomyocytes were treated with 1μM Dox or medium as control for the indicated time. (**A**) Timeframe of (monomeric) phospholamban phosphorylation at the CaMKII specific phosphorylation site Thr17 (p-PLN (Thr17); n = 4) and autophosphorylation of CamKII at Thr286 (p-CaMKII (Thr286); n = 3) after 0-48h Dox treatment. Phosphorylated protein was normalized for total expression of respective protein. (**B**) H_2_O_2_-levels in the cell culture supernatant (n = 3). RLU values were divided by relative cell number. (**C**) SR Ca^2+^ content in control and Dox treated cells after 0-24h. Amplitude and diastolic Ca^2+^ levels determined from Ca^2+^ transients after Dox or medium administration for 0-24h (n = 3). *p < 0.05 for 1-way ANOVA with Bonferroni post hoc testing.

To further enlighten molecular alterations in Dox cardiotoxicity Ca^2+^-dependent signaling and cell death were studied. In NRVCM phosphorylation of phospholamban (PLN) at its CaMKII specific phosphorylation site at Thr17 (after 16-24h) was observed as a result of Dox exposure, coinciding with the peak of p53 accumulation (Figs 1C and 2A). Thr17 phosphorylation of PLN is a sign for enhanced activity of the Ca^2+^/calmodulin activated protein CaMKII. Long-lasting or strong activation of CaMKII is associated with autophosphorylation at Thr 286. Indeed, we were able to detect phosphorylation and autophosphorylation of CaMKII under Dox exposure (Fig 2A). As CaMKII can be activated by either Ca^2+^/calmodulin or reactive oxygen species (ROS), production of H_2_O_2_ at the time of CaMKII activation was assessed. Neither at 16h nor at 24h we could detect elevated H_2_O_2_ formation induced by Dox treatment. As such, Ca^2+^ was the primary suspect for CaMKII activation (Fig 2B).

Therefore, we recorded Ca^2+^ transients in order to detect differences in overall Ca^2+^ handling. Analysis of NRVCM treated with 1μM Dox or medium for up to 24h did not show any differences in diastolic Ca^2+^ content or transient amplitude. But 16h after initiation of Dox treatment—and therefore at the time of CaMKII activation—a significant depletion of SR Ca^2+^ content was observed (Fig 2C).

### Dox induced CaMKII activation and apoptosis are Ca^2+^ dependent

**Fig 3:**
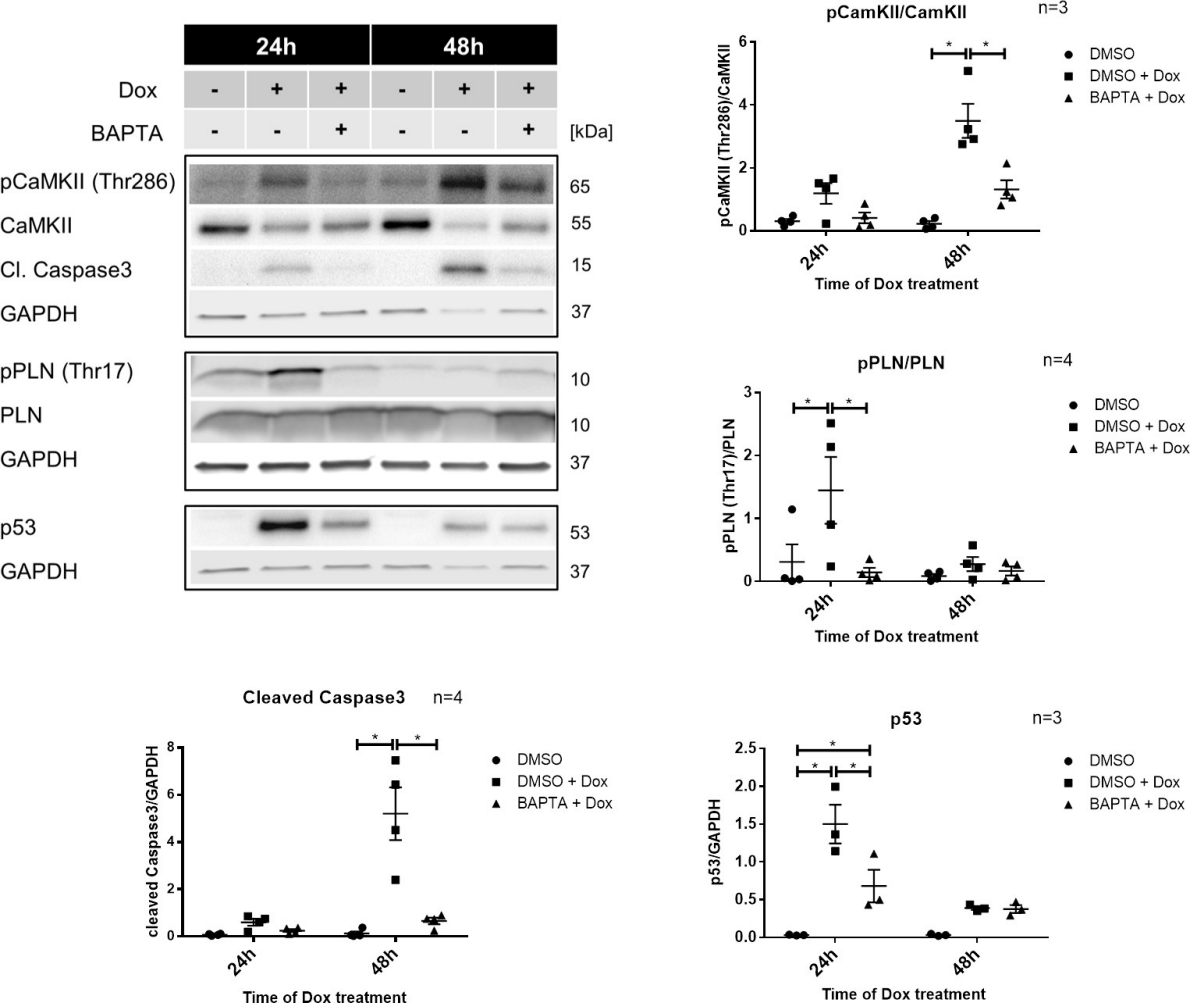
Doxorubicin (Dox) induced Ca^2+^/calmodulin-dependent protein kinase II (CaMKII) activation and apoptosis are Ca^2+^ dependent. Neonatal rat cardiomyocytes were treated with 1μM Dox or medium for 24h or 48h. Extent of (monomeric) phospholamban phosphorylation (pPLN(Thr17); n = 4), CaMKII autophosphorylation pCaMKII(Thr286); n = 4), activation of caspase3 (n = 4) and accumulation of p53 (n = 3) after pretreatment with 20μM BAPTA-AM for 45 min. Glyceraldehyde 3-phosphate dehydrogenase (GAPDH) was used as housekeeping gene. Phosphorylated protein was normalized for total expression of respective protein. *p < 0.05 for 2-way ANOVA with Bonferroni post hoc testing.

Apart from depleted SR Ca^2+^, further evidence that CaMKII activation occurred due to Dox induced alterations in free Ca^2+^ was supported by experiments with the Ca^2+^ chelator BAPTA-AM. In the presence of BAPTA-AM DOX dependent phosphorylation of PLN and CaMKII after 24h or 48h was in fact almost completely abolished, pointing towards Ca^2+^ as main activator (Fig 3). Interestingly, Ca^2+^ chelation by BAPTA-AM did not only reduce CaMKII activation, but also reduced the accumulation of p53 after 24h Dox treatment and completely diminished the levels of cleaved caspase3 (Fig 3 bottom). This clearly indicates that Ca^2+^ is the main trigger for apoptotic signaling pathways activated by Dox in this setting.

### CaMKII activation contributes to Dox toxicity

**Fig 4:**
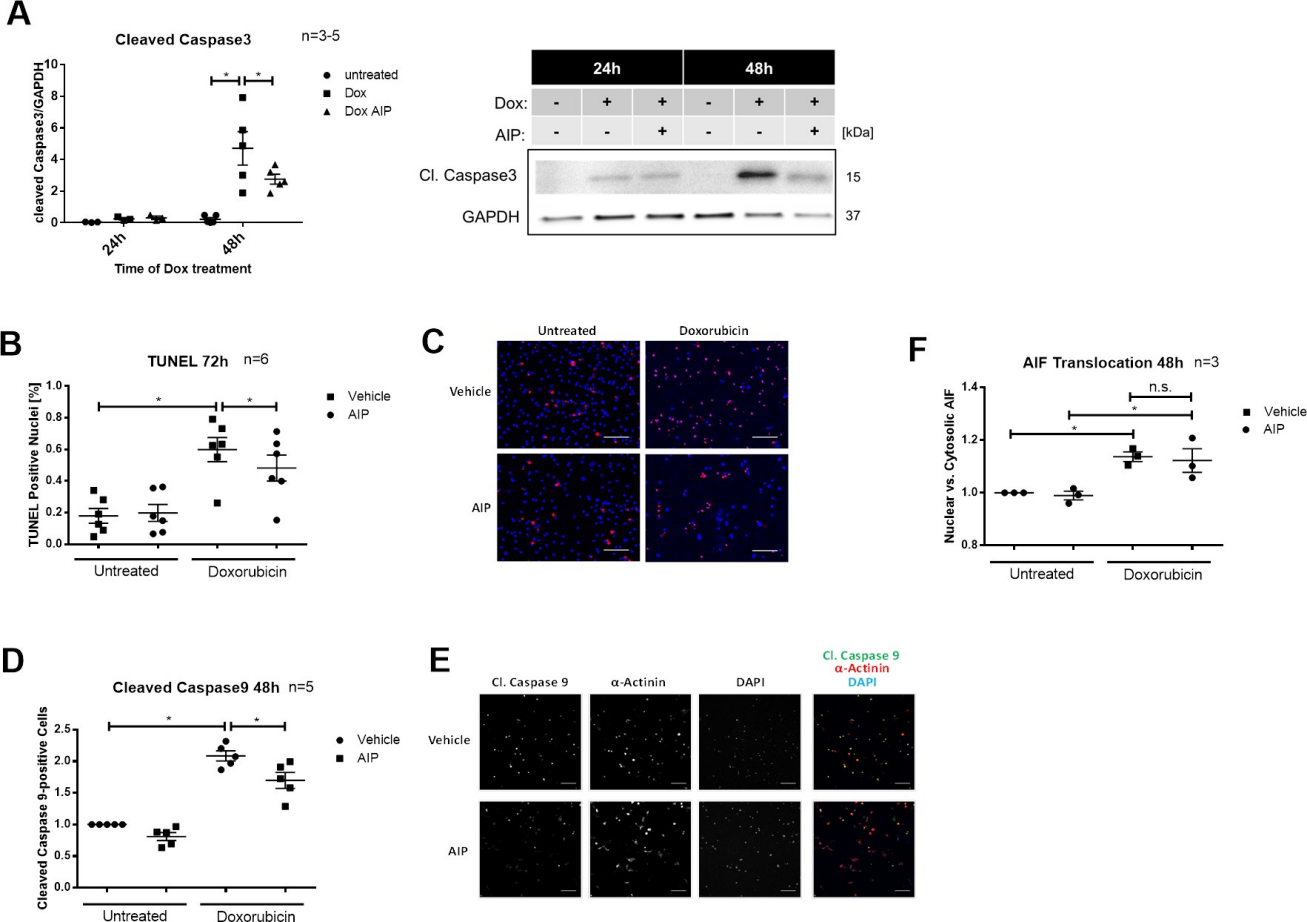
CaMKII activation contributes to Doxorubicin (Dox) cardiotoxicity. Neonatal rat cardiomoycytes were treated with 5μM AIP or vehicle starting 14h after the addition of 1μM Dox or medium (untreated). (**A**) Levels of cleaved caspase3 after 48h Dox treatment (n = 3–5). (**B**) Percentage of TUNEL positive cells in cells treated for 72h with Dox or medium in combination with AIP or vehicle. The ratio of DAPI positive and TUNEL positive nuclei was built. (n = 6). * P < 0.05 for matched 2-way ANOVA with Bonferroni post hoc testing. (**C**) Representative images of TUNEL positive (red) and negative cells. DAPI (blue) was used to stain nuclei. Scale bar represents 100 μm. (**D**) Number of cells positive for cleaved caspase9 after 48h treatment with Dox or ctrl in combination with AIP or vehicle (n = 5) (**E**) Representative images for caspase9 downregulation by AIP treatment. Scale bar represents 100 μm. (**F**) Apoptosis inducing factor (AIF) translocation: Relative signal intensity of anti-AIF antibody in the nucleus vs. cytosol after 48h Dox treatment (with and without AIP inhibition). All images except (B): p < 0.05, **p < 0.01 and ***p < 0.001 all for 1-way ANOVA with Bonferroni post hoc testing.

Interestingly, Dox treatment resulted in CaMKII activation and PLN phosphorylation and increased levels of cleaved caspase3. To investigate this further, we blocked CamKII activity with the specific inhibitor AIP. With 5μM AIP present at the time of highest CaMKII activity apoptosis after 48h Dox treatment—measured by the amount of cleaved caspase3 and the percentage of cells positive for cleaved caspase9—was significantly reduced (Fig 4). Evaluation of late apoptotic events 72h after Dox treatment by TUNEL assay showed a significant reduction of TUNEL positive cells when CaMKII was inhibited by AIP. These results indicate that CaMKII is a contributing factor in Dox cardiotoxicity and is at least partly involved in activation of caspase-dependent apoptosis pathways. On the other hand CAMKII inhibition with AIP in cardiomyocytes treated with Dox for 48h did not have any effect on AIF translocation. In Dox cardiotoxicity the effects of CaMKII therefore seem to be caused mainly by caspase dependent mechanisms (Fig 4)

### GRP78 overexpression in Dox cardiotoxicity modulates CaMKII activity

#### GRP78 overexpression ameliorates Dox cardiotoxicity in a dose dependent manner

So far we could demonstrate that deranged intracellular Ca^2+^ and CaMKII activation are relevant factors in Dox cardiotoxicity. Next we aimed at investigating the potential impact of GRP78 overexpression on these pathways. Therefore we repeated our experiments after transfecting NRVCMs with recombinant AAV6 expressing GRP78 (AAV-GRP78) or Luciferase (AAV-Luc).

**Fig 5:**
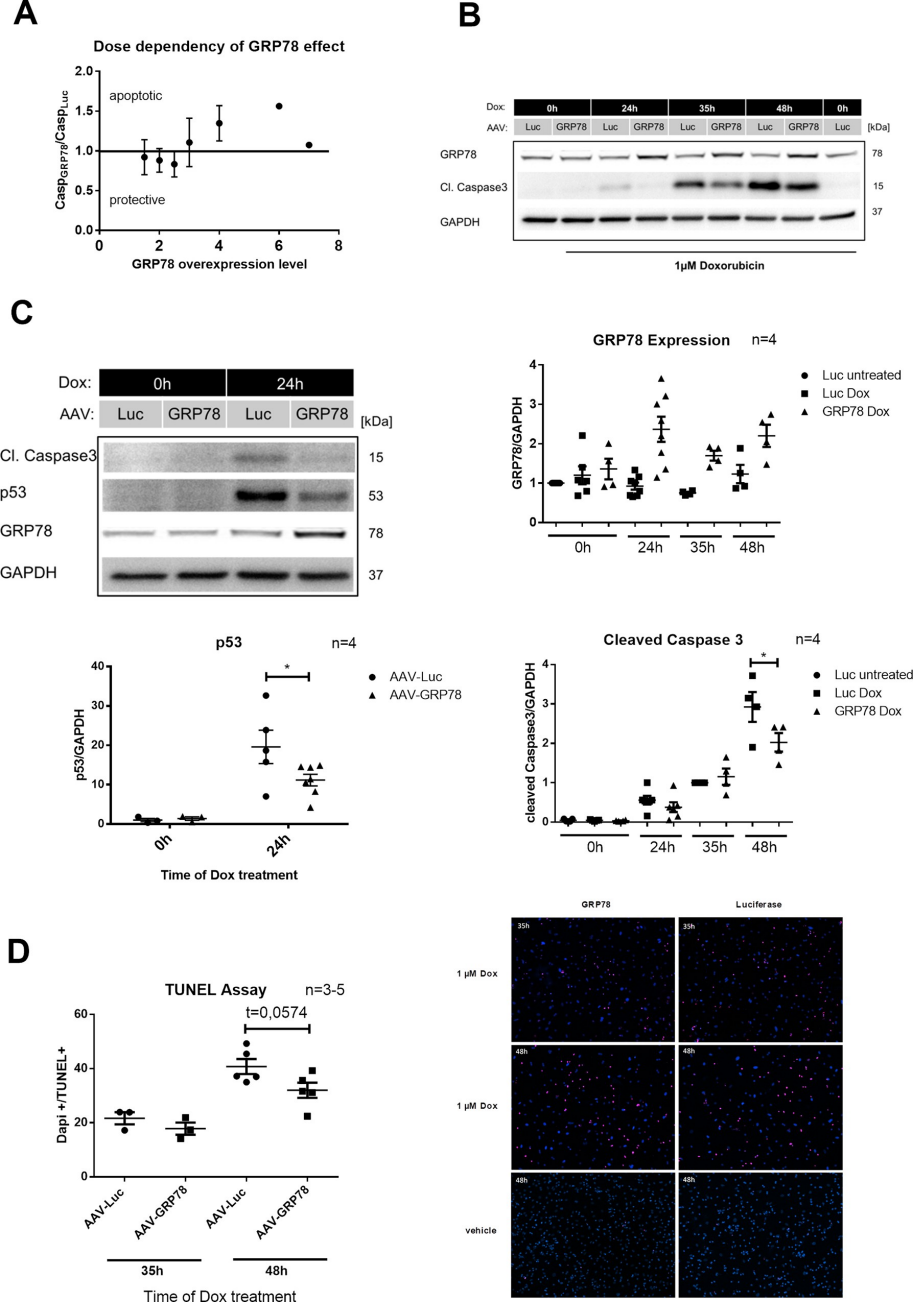
Antiapoptotic effect of glucose regulated protein 78 (GRP78) overexpression after Doxorubicin (Dox) treatment. Neonatal rat cardiomyocytes were transfected with adeno-associated virus 6 expressing GRP78 (AAV-GRP78) or Luciferase (AAV-Luc) two days before treatment with 1μM Dox. (**A**) Protective window of GRP78 overexpression. The ratio of cleaved caspase3 with Luc treatment (Casp_Luc_) and cleaved caspase3 with GRP78 treatment (Casp_GRP78_) was built. Experiments were clustered by GRP78 overexpression and the ratio Casp_Luc_ /Casp_GRP78_ plotted against GRP78 expression clusters (n = 1–12). (**B**) GRP78 protein levels (n = 4) and cleaved caspase3 levels (n = 4) after 0h, 24h, 36h and 48h Dox treatment. (**C**) Dox induced caspase3 cleavage and p53 accumulation with GRP78 overexpression (n = 4). (**D**) Evaluation of apoptosis by the TUNEL Assay. The ratio of DAPI positive and TUNEL positive nuclei was built (n = 3–5). *p < 0.05 for 2-way ANOVA with Bonferroni post hoc testing.

We found that the effect of GRP78 seemed to depend on the extent of overexpression. Low levels of overexpression seemed to be beneficial, while levels higher than a 3 fold overexpression apparently increased caspase3 activation (Fig 5A), revealing a limited therapeutic range for GRP78 expression. For our experiments we consequently excluded samples with GRP78 levels higher than 2.5 fold overexpression (Fig 5B).

Within this therapeutic range GRP78 overexpression decreased Dox induced apoptosis. Dox induced accumulation of p53 after 24h and most importantly caspase3 cleavage after 48h were significantly reduced (Fig 5B and 5C). In support of this finding, detection of apoptotic nuclei by TUNEL Assay (Roche, Mannheim, Germany) also showed a tendency (t = 0.05 for direct comparison) towards reduced apoptosis with GRP78 overexpression after 24h Dox treatment (Fig 5D). The smaller effect with this method likely reflects the fact that the events measured by TUNEL assay occur later than caspase3 cleavage.

### GRP78 reduces CaMKII activation

**Fig 6:**
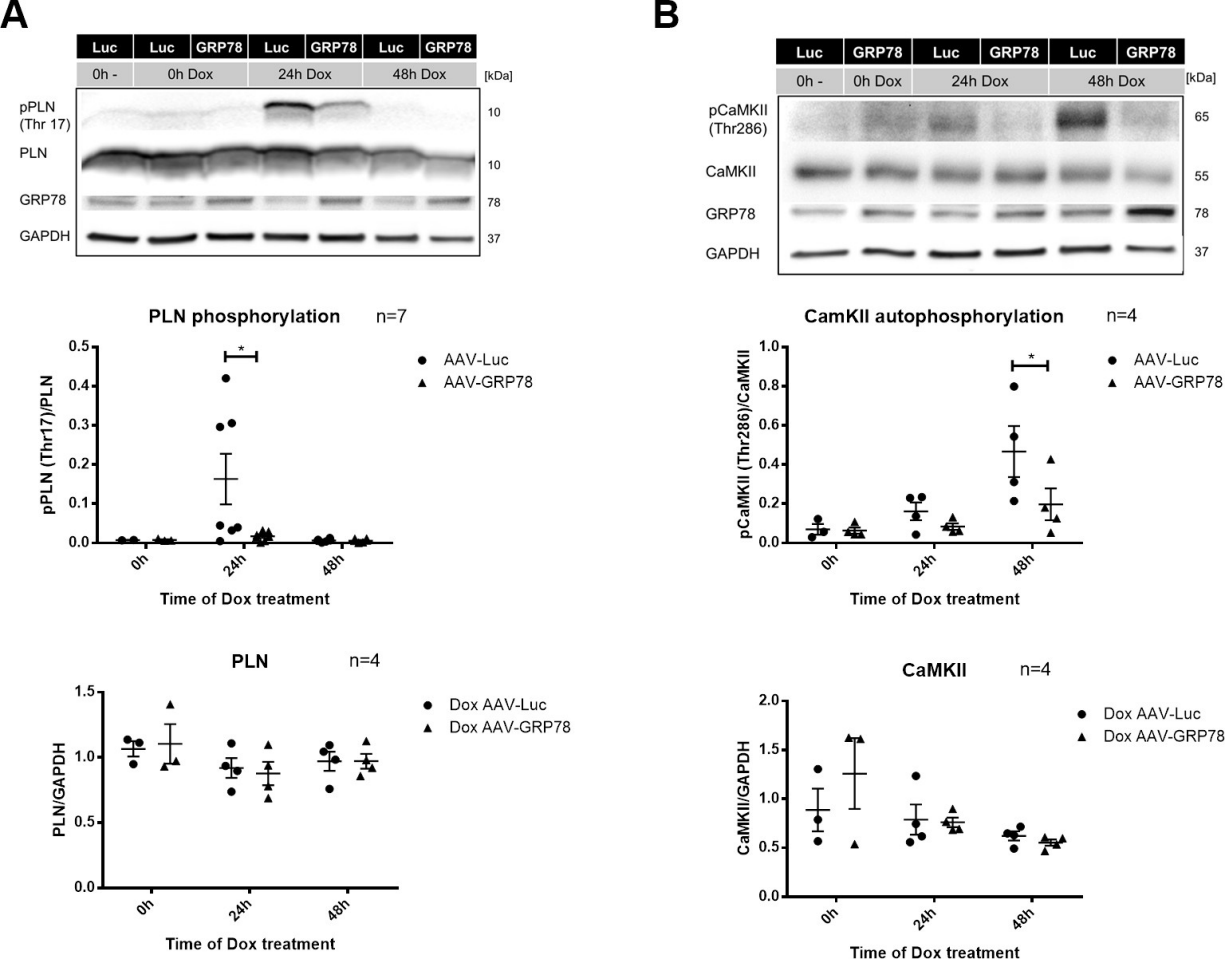
Ca^2+^/calmodulin-dependent protein kinase II (CaMKII) activity and phosphorylation after doxorubicin (Dox) treatment with glucose regulated protein 78 (GRP78) overexpression. Neonatal rat cardiomyocytes were transfected with adeno-associated virus 6 expressing GRP78 (AAV-GRP78) or Luciferase (AAV-Luc) two days prior to treatment with 1μM Dox. (**A**) Dox induced time dependent changes in (monomeric) phospholamban (PLN) levels (n = 4) and phosphorylation at Thr17 with AAV-GRP78 treatment (n = 7). (**B**) Time dependent changes in CaMKII levels (n = 4) and autophosphorylation at Thr286 after Dox treatment (n = 4). Glyceraldehyde 3-phosphate dehydrogenase (GAPDH) was used as housekeeping gene. Phosphorylated protein was normalized for total expression of respective protein *p < 0.05 for 2-way ANOVA with Bonferroni post hoc testing.

Our results described above indicate that CaMKII contributes to Dox cardiotoxicity and that free Ca^2+^ is an important factor in this pathway, that itself promotes cardiotoxicity. GRP78 is known to modulate ER Ca^2+^ homeostasis[7]. Therefore, we explored whether GRP78 exerts its protective effects by alterations in Ca^2+^/CaMKII related pathways. Indeed, Dox induced phosphorylation of PLN after 24h was almost completely abolished in AAV-GRP78 treated NRVCMs when compared to the AAV-Luc treated controls (Fig 6A). Furthermore, CaMKII autophosphorylation after 48h was also significantly decreased by GRP78 overexpression (Fig 6B). In this regard, GRP78 treatment inhibits Dox induced CaMKII activation.

#### Mice treated with AAV-GRP78 show improved cardiac function and survival

*In vitro* GRP78 overexpression protects cardiomyocytes from apoptosis and changes in Ca^2+^/CaMKII signaling induced by Dox treatment. Additional experiments were therefore conducted to investigate the protective potential of cardiac GRP78 overexpression in our *in vivo* model of chronic Dox cardiotoxicity.

**Fig 7:**
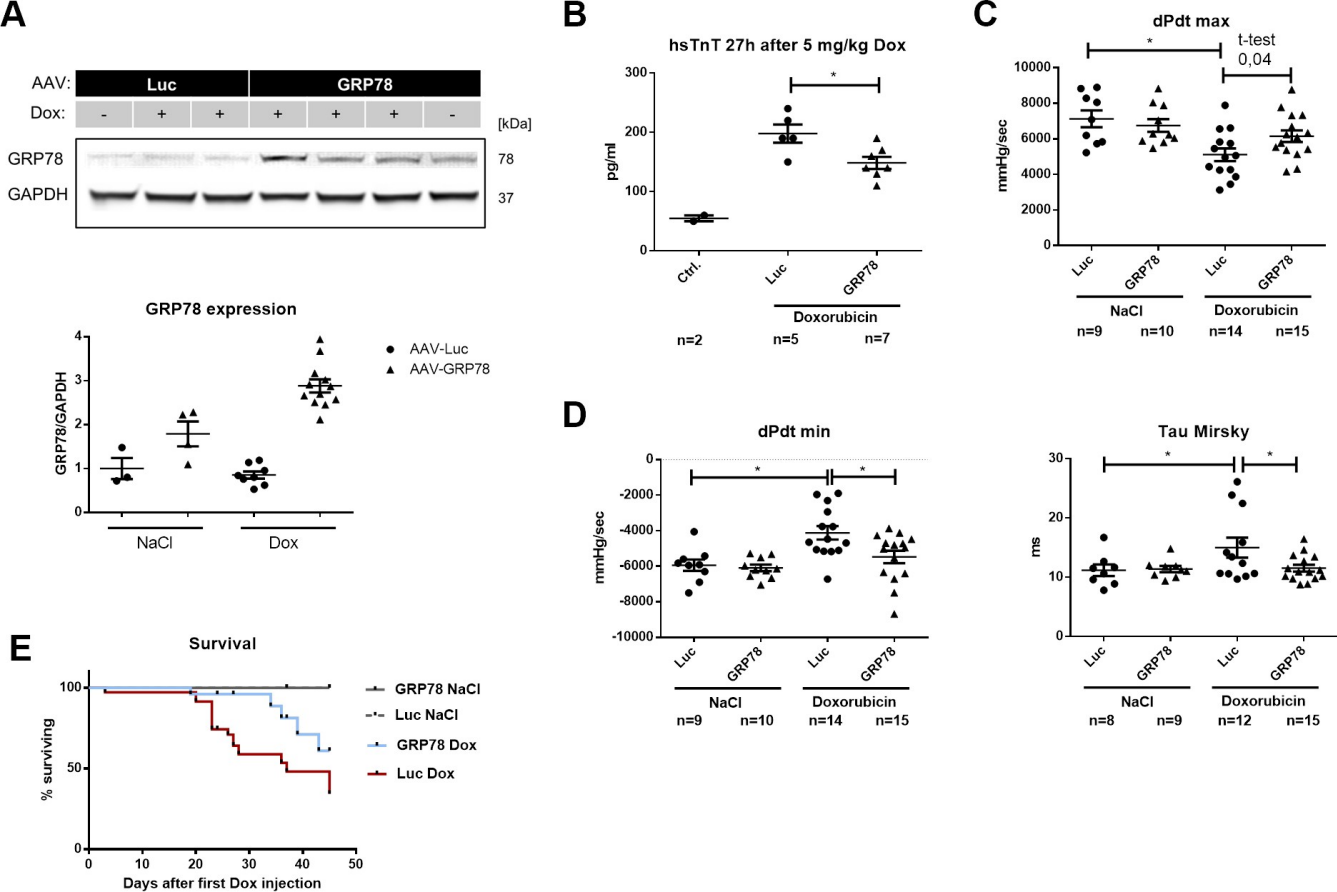
Effect of glucose regulated protein 78 (GRP78) genetransfer on cardiac function and survival *in vivo*. Male C57/Bl6N were transfected with 2.5 *10^12^ viral genomes of a virus expressing GRP78 or Luciferase (Luc). Then they were injected with a cumulative Doxorubicin (Dox) dose of 20 mg/kg or NaCl over 3 weeks. (**A**) GRP78 expression 8 weeks after transfection. Glyceraldehyde 3-phosphate dehydrogenase (GAPDH) was used as housekeeping gene. Luc NaCl n = 3; GRP78 NaCl n = 4; Luc Dox n = 8; GRP78 Dox n = 12. (**B**) Plasma high sensitive Troponin T (hsTnT) levels 27h after the last Dox injection for determination of cardiomyocyte death (n for each group is depicted in the Figure). (**C**) Catheter based intraventricular assessment of minimal (dP/dtmin) pressure velocity as parameter for systolic function (n for each group is depicted in the Figure). (**D**) Catheter based assessment of diastolic ventricular function. Dox induced changes in minimal (dP/dtmin) and maximal (dP/dtmax) pressure velocity and relaxation parameter Tau Mirsky (n for each group is depicted in the Figure). *p < 0.05 for 1-way ANOVA with Bonferroni post hoc testing. (**E**) Overall survival after Dox treatment in all groups. Luc NaCl n = 10; GRP78 NaCl n = 10; Luc Dox n = 29; GRP78 Dox n = 25. *Log-rank (Mantel-Cox) Test t (P = 0.023).

Transfection with AAV9 expressing GRP78 (AAV-GRP78) resulted in a 2–3 fold overexpression of GRP78 in left ventricular myocardial tissue (Fig 7A). Interestingly, expression in the Dox treated group was higher than in the saline treated group, which matches earlier observations that Doxorubicin enhances expression from the CMV promoter/enhancer.[9] In order to determine acute cardiac damage induced by Dox we used high-sensitive troponin T (hsTnT) plasma levels 27h after the last Dox injection. A 4 fold rise of plasma hsTnT levels in the AAV-Luc transfected and Dox injected group indicated a massive decline of cardiomyocytes (Fig 7B). In analogy to our *in vitro* experiments a protective effect on cardiomyocyte viability could be shown directly with significantly reduced plasma hsTnT levels in the AAV-GRP78 group.

Assessment of cardiac function by echocardiography 3 weeks later did not show any difference in ejection fraction (see S1 Fig). The more sensitive intraventricular pressure-volume (PV)-loop analysis revealed beginning cardiac dysfunction with impaired minimal (dP/dtmin) and maximal pressure velocity (dP/dtmax) as well as reduced peak pressures in Dox/AAV-Luc treated animals (Fig 7C and 7D, peak pressures see S2 Fig). Additionally, a 2 fold rise of the parameter Tau Mirsky in AAV-Luc treated animals indicated impaired relaxation after Dox treatment (Fig 7D). GRP78 overexpression revealed a small trend towards improvement with regards to systolic cardiac function represented by dP/dtmax. Interestingly, a strong protective effect was induced in diastolic function. Minimal pressure velocity and relaxation (Tau) were almost restored to normal values (Fig 7D) by GRP78 overexpression in Dox treated mice.

Most importantly, the protective effect of GRP78 overexpression resulted in improved overall survival. 45 days after the initiation of Dox treatment 62% of the AAV-Luc treated mice had died compared to only 39% in the AAV-GRP78 group (Fig 7E).

## Discussion

Anticancer treatment has made considerable progress over the last 20 years. Prolonged survival now poses new challenges, as many chemotherapeutics–Dox being one of the most prominent–impose cardiotoxic side-effects. Still the underlying mechanisms for Dox cardiotoxicity are not fully understood and current therapeutic recommendations are mainly symptomatic. Our results shed new light on Dox induced alterations in Ca^2+^ handling for the induction of cardiomyocyte apoptosis. To our knowledge this is the first time apoptotic effects of Ca^2+^ induced CaMKII activity have been explored as a consequence of Dox treatment. Furthermore, we have direct evidence that therapeutic overexpression of the ER chaperone GRP78 partly protects from Dox induced cardiotoxicity by reducing Dox induced CaMKII activation.

### CaMKII and the role of Ca^2+^ in dox cardiotoxicity

In the past cardiotoxic effects of Dox have been explored using a variety of models and doses (ranging from 0.01μM -100μM) which might lead to different pathways being activated [10]. Accordingly, many stressors are being associated with Dox cytotoxicity. Studies in patients undergoing Dox chemotherapy have reported plasma peak levels between 0.5μM—1μM followed by a plateau of 0.05μM Dox [11, 12]. Our models in this current study therefore are comparable to the clinical situation.

During the time course of Dox induced apoptosis we observed the activation of the cell death promoter p53 and Ca^2+^ sensitive proteins like PLN and CaMKII. Interestingly, most alterations occurred 16h after initiation of Dox treatment. At this time point SR Ca^2+^ content is significantly diminished in Dox treated cells. Depletion of SR Ca^2+^ content is consistent with observations in other *in vitro* models of Dox cardiotoxicity [3, 13]. It is thought to be the result of an SR Ca^2+^ leak induced by interaction of Dox with the ryanodine receptor (RyR) and the sarco/endoplasmic reticulum Ca^2+-^ATPase (SERCA2A): While Dox doses from 0.01μM-2.5μM activate the RyR by direct ligand binding, it is suggested that Ca^2+^ re-uptake into the SR is inhibited by redox modification of SERCA2A by the Dox metabolite doxorubicinol [14]. Other known factors contributing to an enhanced open probability of the RyR after Dox treatment are modifications by ROS and phosphorylation by CaMKII [3, 14]. We could not detect elevated formation of H_2_O_2_ at the time of SR Ca^2+^ depletion. While this does not rule out the possibility, that other ROS might be elevated and therefore contribute to SR Ca^2+^ depletion, we did see PLN phosphorylation at Thr17—a sign of enhanced activity of CaMKII.

It is striking that PLN phosphorylation decreases after 48h, the timepoint when CaMKII phosphorylation at Thr286 is the strongest. This discrepancy can be explained by the fact that PLN phosphorylation at Thr is controlled not only by CaMKII but also by phosphatases like calcineurin, which catalyze dephosphorylation. A similar effect as with our experiments was seen in human heart failure, where reduced PLN phosphorylation with high CamKII activity could be linked to increased calcineurin activity [15, 16]. Both PLN and CaMKII activation were almost completely abolished with elimination of free cytosolic Ca^2+^ by BAPTA-AM. CaMKII activation therefore seemed to be the result and not the main inductor of the SR Ca^2+^ leak in our model. On the other hand SR Ca^2+^ leak seems to be influenced by CaMKII activity [17]. Indeed, Sag et al came to a similar conclusion observing CaMKII dependent impairment of Ca^2+^ handling and SR Ca^2+^ leak in Dox treated cardiomyocytes [3].

So far the role of CaMKII in Dox cardiotoxicity has scarcely been investigated. However, in several other heart failure models it was recently shown that CaMKII regulates cell death by promoting Ca^2+^ flux to the mitochondria and accumulation of p53 [18, 19]. In our experiments CaMKII activity correlated with the extent of Dox induced caspase3 activation. More importantly, inhibition of CaMKII by AIP reduced caspase3 cleavage and the amount of cells positive for cleaved caspase 9 after 48h. Also the percentage of TUNEL positive cells after 48h Dox treatment decreased when CaMKII was inhibited. The effect was smaller than the effect on both caspase isoforms, which most likely reflects the fact that the events measured by TUNEL assay occur later than caspase3 or caspase9 cleavage. Similarly we also only saw a tendency towards reduced apoptosis with TUNEL Assay after 48h in our experiments with GRP78 overexpression. To our knowledge these are the first data directly linking CaMKII to Dox induced cell death. Apart from caspase mediated apoptosis, a relevant part of Dox induced toxicity is induced by AIF and therefore independently from caspase activation [20]. We too saw translocation of AIF with Dox treatment, but it was not changed by CaMKII inhibition. This indicates that in Dox cardiotoxicity, CaMKII is involved in caspase dependent apoptosis pathways, but not caspase3 independent cell death.

Additionally, effects seen with the elimination of free Ca^2+^ by BAPTA-AM in our experiments not only suggest that Dox induced PLN and CaMKII phosphorylation are strongly Ca^2+^ dependent. Furthermore, the accumulation of p53 and the activation of caspase3 to a certain extent depend on free Ca^2+^. CaMKII activation therefore seems to be one part in a bigger network of effects triggered by Dox induced increase of calcium levels.

### Protective effects of GRP78

The main aim of this study was to further explore possible protective mechanisms of GRP78 overexpression in Dox cardiotoxicity. In our NRVCM model gene transfer with full-length GRP78 including the KDEL and the ER motif did diminish caspase3 activation.

Membrane localized GRP78 is believed to be one way of conferring protection from chemotherapeutics in tumor cells via the activation of the protective PI3K-AKT pathway [21]. GRP78 induced by ER stress is known to phosphorylate AKT directly on Ser473 [22]. Furthermore, it was previously shown that levels of membrane localized GRP78 rise with ER Stress and ectopic GRP78 overexpression. Nevertheless, enhanced AKT phosphorylation surprisingly is not the protective mechanism in our experiments, as Dox induced AKT phosphorylation was diminished with protective GRP78 overexpression (see S4 Fig). Thus, in our model membrane bound GRP78 is not likely to play a role in reducing Dox toxicity. We saw that Dox itself induced AKT phosphorylation, which is a secondary response to cell death inducing stressors including calcium [23]. The reduced AKT phosphorylation we observed with GRP78 overexpression can thus be explained by a reduced stress response.

Interestingly, CaMKII activation and p53 accumulation were significantly reduced concomitantly to diminished Caspase3 levels by GRP78 overexpression. As both pathways are Ca^2+^ dependent and influence apoptosis, a possible explanation for the beneficial action of GRP78 was normalization of Ca^2+^ handling. Recently, a protective effect of GRP78 overexpression on Dox treated cardiomyocytes *in vitro* and *in vivo* was linked to the ability of GRP78 to restore a protective ER stress response, which is disturbed by Dox [24]. In contrast to our results, Fu et al did not see a reduction in GRP78 protein levels with Dox treatment, but only a failure of Xbp-1 mediated induction of protective GRP78 expression. We found that the use of GRP78 antibodies binding to different epitopes resulted in different expression patterns. This discrepancy might therefore reflect differing responses of GRP78 expression in different compartments (for example ER vs. cytosol). Nonetheless our experiments confirm the beneficial effect of GRP78 overexpression on Dox induced apoptosis and add another layer to these results: SR Ca^2+^ homeostasis and ER stress are closely interlinked, therefore SR calcium depletion and activation of CaMKII as seen in our experiments may be a result of or enhanced by Dox induced ER stress [25]. Other factors, that are currently discussed as factors contributing to Dox cardiotoxicity, are for example ATP-shortage due to reduced oxidative phosphorylation, increased ROS production, and most importantly, inhibition of Topoisomerase II [26–29]. A common factor of the first two factors is mitochondrial stress and dysfunction, which is also influenced by CaMKII activity and ER stress: Both ER stress and increased CaMKII activity have been shown to elevate mitochondrial Ca^2+^-level, leading to mitochondrial stress, ROS production and membrane transition pore opening [18, 30]. So far we have only examined ROS production for time points up to 24h, in order to rule out ROS as initial activators of CaMKII. It is tempting to speculate that secondary ROS production and ensuing oxidative modification of CaMKII, which renders CamKII completely independent of Ca^2+^, might be mechanisms further enhancing cardiomyocyte death and dysfunction. Possible effects of Dox induced CaMKII activation on mitochondrial function and ROS production will be the subject of future experiments.

### Are these results relevant for *in vivo* cardiotoxicity?

Apoptosis is known to be a sign of acute Dox cardiotoxicity *in vivo* [8]. In our *in vivo* model a protective effect of GRP78 is directly reflected by reduced levels of hsTnT 72h after Dox treatment indicating reduced decline of cardiomyocytes. In this regard, as well as the reduction but not complete inhibition of p53 accumulation, GRP78 treatment very closely resembles the effect of BAPTA-AM that we saw *in vitro*.

Long-term cardiotoxic effects of Doxorubicin can lead to declining cardiac function and heart failure [31]. Diastolic dysfunction was previously established as an early and important event in Dox cardiomyopathy, predicting later systolic dysfunction [32, 33]. Interestingly, we observed improved diastolic function with GRP78 overexpression *in vivo*, which could be of particular relevance. The effect of GRP78 overexpression on diastolic dysfunction was more pronounced than the effect on systolic dysfunction.

Diastolic dysfunction can occur due to impaired calcium handling, myofilament function and organization or impaired cardiomyocyte energetics [34]. Also, increased fibrosis influences relaxation properties [35]. The attenuating effect of GRP78 on diastolic dysfunction may therefore be a result of the reduced decline of cardiomyocytes observed with GRP78 overexpression and/or reduced maladaptive cardiac remodeling (which is influenced by CaMKII among other factors [36]). On the other hand long-lasting effects on calcium handling may be induced by GRP78 overexpression. Therefore further in depth evaluation of modulation Ca^2+^ handling and CaMKII by GRP78 is planned in future studies.

## Conclusion

In conclusion, we could provide first evidence that Dox induced Ca^2+^ dependent CaMKII activation directly contributes to Dox cardiotoxicity. Furthermore, we found that GRP78 overexpression reduces the activation of CaMKII and thereby influences proapoptotic and Ca^2+^ dependent pathways involved in Dox cardiotoxicity. GRP78 treatment translates into prolonged survival as well as improved diastolic and to a certain extent systolic cardiac function in our mouse model of Dox cardiotoxicity. As such GRP78 gene therapy holds potential for prophylactic protection from cardiotoxic effects of Dox.

## Supporting information

S1 Fig Effect of glucose regulated protein 78 (GRP78) genetransfer on endsystolic and maximum pressure *in vivo*. Male C57/Bl6N were transfected with 2.5 *10^12^ viral genomes of a virus expressing GRP78 or Luciferase (Luc). Then they were injected with a cumulative doxorubicin (Dox) dose of 20 mg/kg or NaCl over 3 weeks. Shown are Dox induced changes in endsystolic pressure (ESP) and maximum pressure (Pmax) as analyzed by catheter based intraventricular assessment. *P< 0.05 for 1-way ANOVA with Bonferroni post hoc testing. For ESP: Luc NaCl n = 9; GRP78 NaCl n = 10; Luc Dox n = 13; GRP78 Dox n = 13. For Pmax: Luc NaCl n = 3; GRP78 NaCl n = 5; Luc Dox n = 10; GRP78 Dox n = 9.
(TIF)

S2 Fig Echocardiographic assessment of cardiac function *in vivo*. Male C57/Bl6N were transfected with 2.5 *10^12^ viral genomes of a virus expressing GRP78 or Luciferase (Luc). Then they were injected with a cumulative doxorubicin (Dox) dose of 20 mg/kg or NaCl over 3 weeks. Echocardiography was performed 2–3 after the last injection. (**a**) Shown are fractional shortening as determined by echocardiography (FS Echo) and ejection fraction measured by catheter based intraventricular assessment (EF PV-Loop). Echo: Luc NaCl n = 5; GRP78 NaCl n = 5; Luc Dox n = 12; GRP78 Dox n = 14. PV-Loop: Luc NaCl n = 8; GRP78 NaCl n = 10; Luc Dox n = 14; GRP78 Dox n = 15. (**b**) Representative echocardiography pictures from one mouse of each group as used for evaluation of fractional shortening.
(TIF)

S3 Fig Effect of Doxorubicin injection on GRP78. (A) GRP78 mRNA and protein level 24h after i.p.-injection of 20 mg/kg Doxorubicin (Dox) or NaCl (untreated). (B) GRP78 mRNA and protein level 5 days after i.p.-injection of 25 mg/kg Doxorubicin (Dox) or NaCl (untreated). GRP78 levels are significantly decreased. Expression of GRP78 was normalized to HPRT1 mRNA or GAPDH protein. Statistic: unpaired, two-tailed t-Test (* *P*<0.05).
(TIF)

S4 Fig Effect of GRP78 overexpression on Doxrorubicin-induced AKT-phosphorylation. Isolated ventricular cardiomyocytes were transduced with AAV.GRP78 or AAV.Luc and treated with vehicle (untreated) or 1 μM Doxorubicin for 24h and 48h. AKT (Ser473) is significantly phosphorylated after 48h of Dox-treatment, which is diminished by GRP78-overexpression. *P<0.05 for 1-way ANOVA with Bonferroni post hoc testing.
(TIF)

S5 Fig Effect of AIP on Doxorubicin-induced PLN-phosphorylation. Isolated ventricular cardiomyocytes were treated with vehicle (untreated) or 1 μM Doxorubicin for 24h and 48h. Dox induced significant p(Thr17)-PLN Phosphorylation after 24 h, which was moderately diminished by AIP. *P<0.05 for 1-way ANOVA with Bonferroni post hoc testing.
(TIF)

S6 Fig Effect of Doxorubicin injection on CaMKII-Phosphorylation. 24h after injecting mice with 20 mg/kg Doxorubicin (Dox) or NaCl (untreated), left ventricular myocardium was used to perform immunoblot (bottom panel). *P<0.05 for Mann-Whitney test. n = 3 (untreated)– 6 (Dox-treated)
(TIF)

S7 Fig Original immunoblots shown in Fig 1A, 1B and 1C. Immunoblots were stained for KDEL and GAPDH (Fig 1A and 1B) or p53, Cleaved Caspase 3 and GAPDH (Fig 1C). St: Sample used for blot-to-blot normalization.
(TIF)

S8 Fig Original immunoblot shown in Fig 2. Immunoblot was stained for p-CaMKII (Thr286), CaMKII, pPLN (Thr17), PLN and GAPDH. St: Sample used for blot-to-blot normalization.
(TIF)

S9 Fig Original immunoblot shown in the top immunoblot panel of Fig 3. Immunoblot was stained for p-CaMKII (Thr286), CaMKII, Cleaved Caspase 3 and GAPDH. St: Sample used for blot-to-blot normalization.
(TIF)

S10 Fig Original immunoblots shown in the middle (blot 2) and bottom (blot 3) immunoblot panel of Fig 3. Immunoblots were stained for pPLN (Thr17), PLN and GAPDH (blot 2) or p53 and GAPDH (blot 3). St: Sample used for blot-to-blot normalization.
(TIF)

S11 Fig Original immunoblot shown in Fig 4A. Immunoblot was stained for Cleaved Caspase 3 and GAPDH. St: Sample used for blot-to-blot normalization.
(TIF)

S12 Fig Original immunoblot shown in Fig 5B. Immunoblot was stained for GRP78/BiP, GAPDH and Cleaved Caspase 3.
(TIF)

S13 Fig Original immunoblot shown in Fig 5C. Immunoblot was stained for GRP78/BiP, p53, Cleaved Caspase 3 and GAPDH. St: Sample used for blot-to-blot normalization.
(TIF)

S14 Fig Original immunoblot shown in Fig 6A. Immunoblot was stained for GRP78/BiP, pPLN (Thr17), PLN and GAPDH. St: Sample used for blot-to-blot normalization.
(TIF)

S15 Fig Original immunoblot shown in Fig 6B. Immunoblot was stained for p-CaMKII (Thr286), CaMKII, GRP78/BiP and GAPDH. St: Sample used for blot-to-blot normalization.
(TIF)

S16 Fig Original immunoblot shown in Fig 7A. Immunoblot was stained for GRP78/BiP and GAPDH. St: Sample used for blot-to-blot normalization.
(TIF)

S17 Fig Original immunoblots shown in S3 Fig. **A and B.** Immunoblots were stained for GRP78/BiP and GAPDH. For visualization of GRP78 expression, densitometric quantification was added under each band. The ratio of GRP78 to GAPDH is added under the immunoblot.
(TIF)

S18 Fig Original immunoblots shown in S4 Fig. Immunoblots were stained for P-AKT (Ser473), total AKT and GAPDH. St: Sample used for blot-to-blot normalization.
(TIF)

S19 Fig Original immunoblot shown in S6 Fig. Immunoblot was stained for p-CaMKII (Thr286) and CaMKII.
(TIF)

S1 File Expanded materials and methods section. (DOCX)

S2 File Tabular summary of all data included in the manuscript. (XLSX)
