# Supplementary figures and images for: CaMKII activation participates in doxorubicin cardiotoxicity and is attenuated by moderate GRP78 overexpression
Source: PLoS One. 2019 Apr 29;14(4):e0215992. doi: 10.1371/journal.pone.0215992 (PMC6488194; doi:10.1371/journal.pone.0215992)

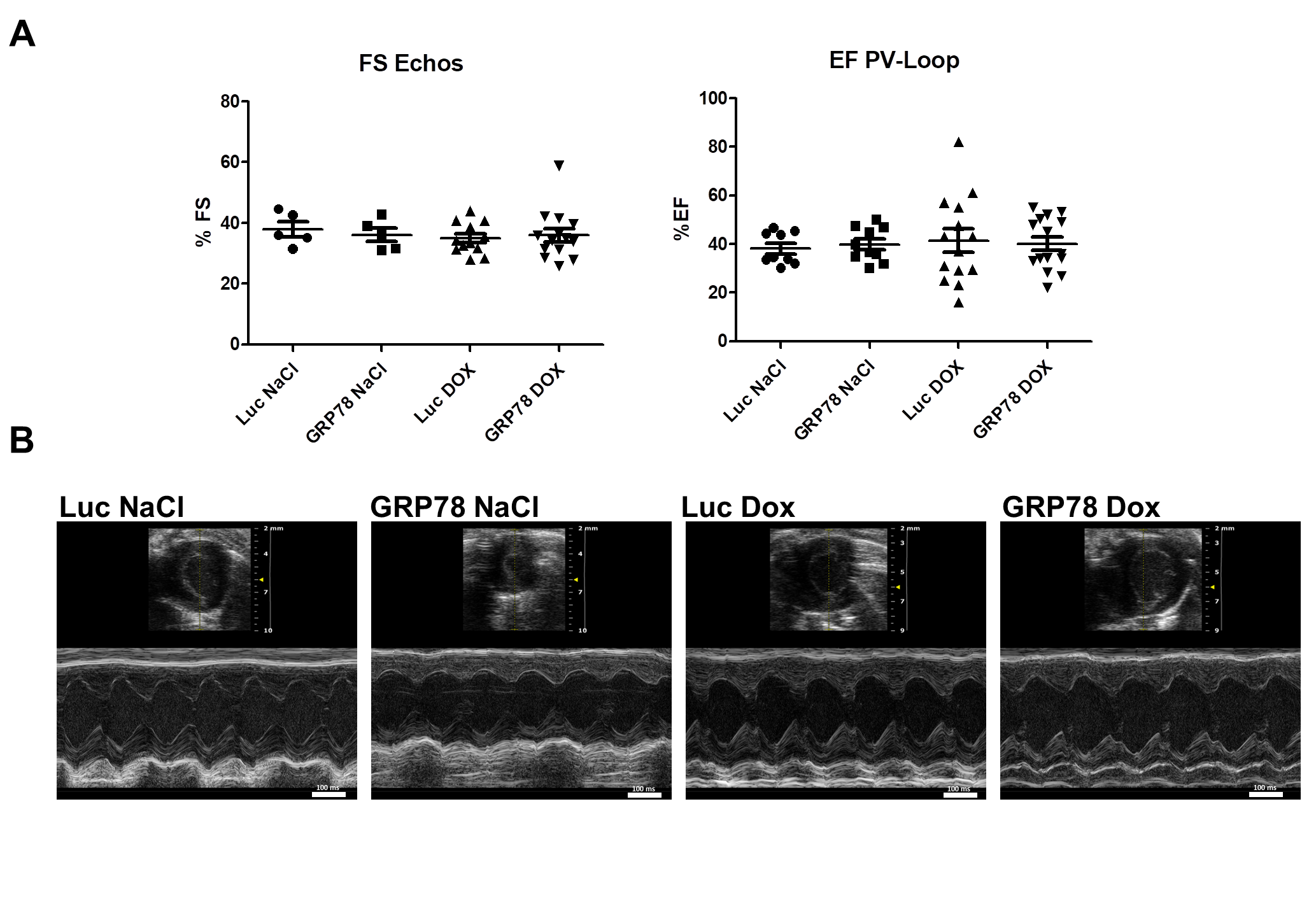

Supplement: S1 Fig — Male C57/Bl6N were transfected with 2.5 *1012 viral genomes of a virus expressing GRP78 or Luciferase (Luc). Then they were injected with a cumulative doxorubicin (Dox) dose of 20 mg/kg or NaCl over 3 weeks. Shown are Dox induced changes in endsystolic pressure (ESP) and maximum pressure (Pmax) as analyzed by catheter based intraventricular assessment. *P< 0.05 for 1-way ANOVA with Bonferroni post hoc testing. For ESP: Luc NaCl n = 9; GRP78 NaCl n = 10; Luc Dox n = 13; GRP78 Dox n = 13. For Pmax: Luc NaCl n = 3; GRP78 NaCl n = 5; Luc Dox n = 10; GRP78 Dox n = 9. (TIF) [file pone.0215992.s001.TIF]

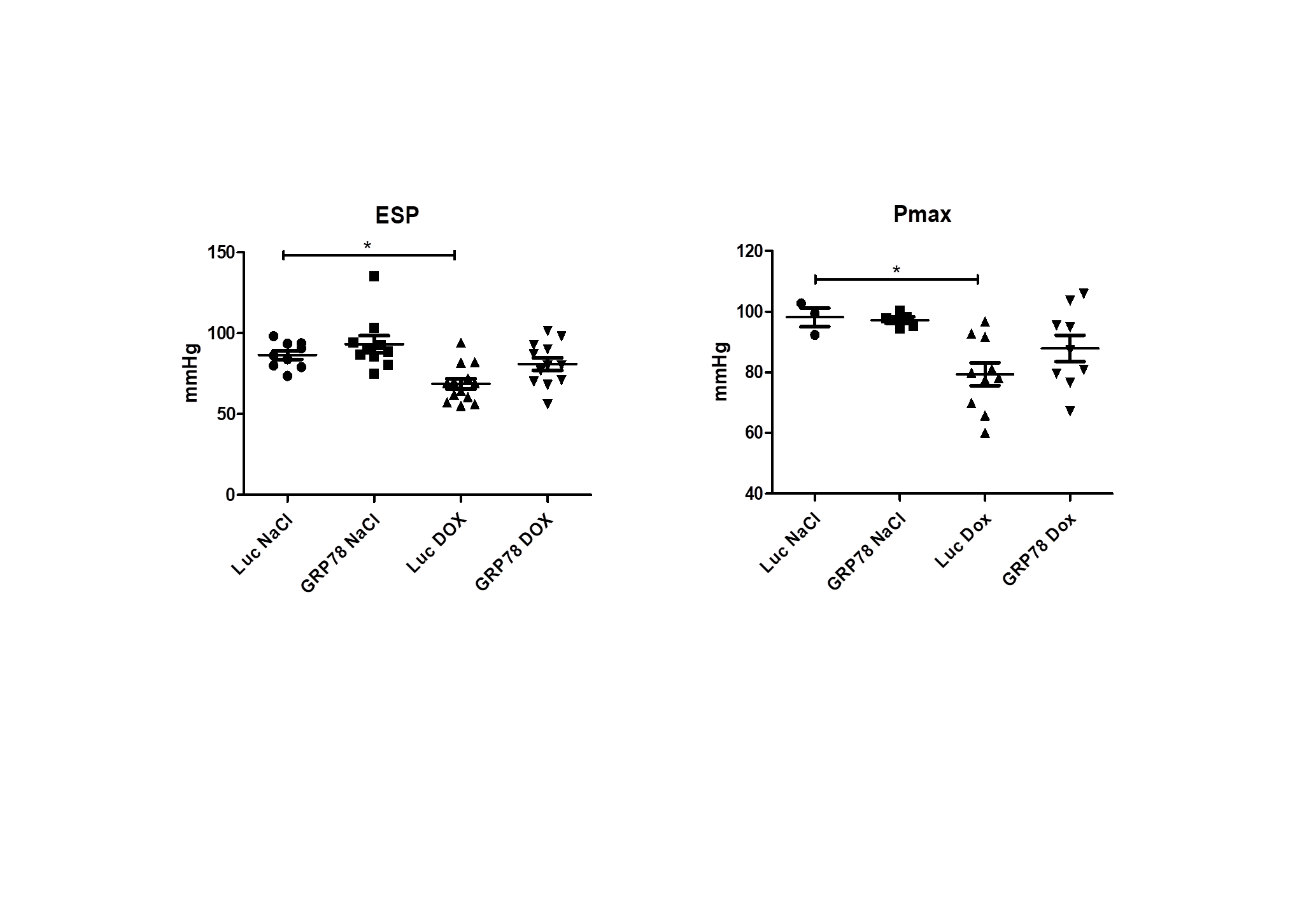

Supplement: S2 Fig — Male C57/Bl6N were transfected with 2.5 *1012 viral genomes of a virus expressing GRP78 or Luciferase (Luc). Then they were injected with a cumulative doxorubicin (Dox) dose of 20 mg/kg or NaCl over 3 weeks. Echocardiography was performed 2–3 after the last injection. (a) Shown are fractional shortening as determined by echocardiography (FS Echo) and ejection fraction measured by catheter based intraventricular assessment (EF PV-Loop). Echo: Luc NaCl n = 5; GRP78 NaCl n = 5; Luc Dox n = 12; GRP78 Dox n = 14. PV-Loop: Luc NaCl n = 8; GRP78 NaCl n = 10; Luc Dox n = 14; GRP78 Dox n = 15. (b) Representative echocardiography pictures from one mouse of each group as used for evaluation of fractional shortening. (TIF) [file pone.0215992.s002.TIF]

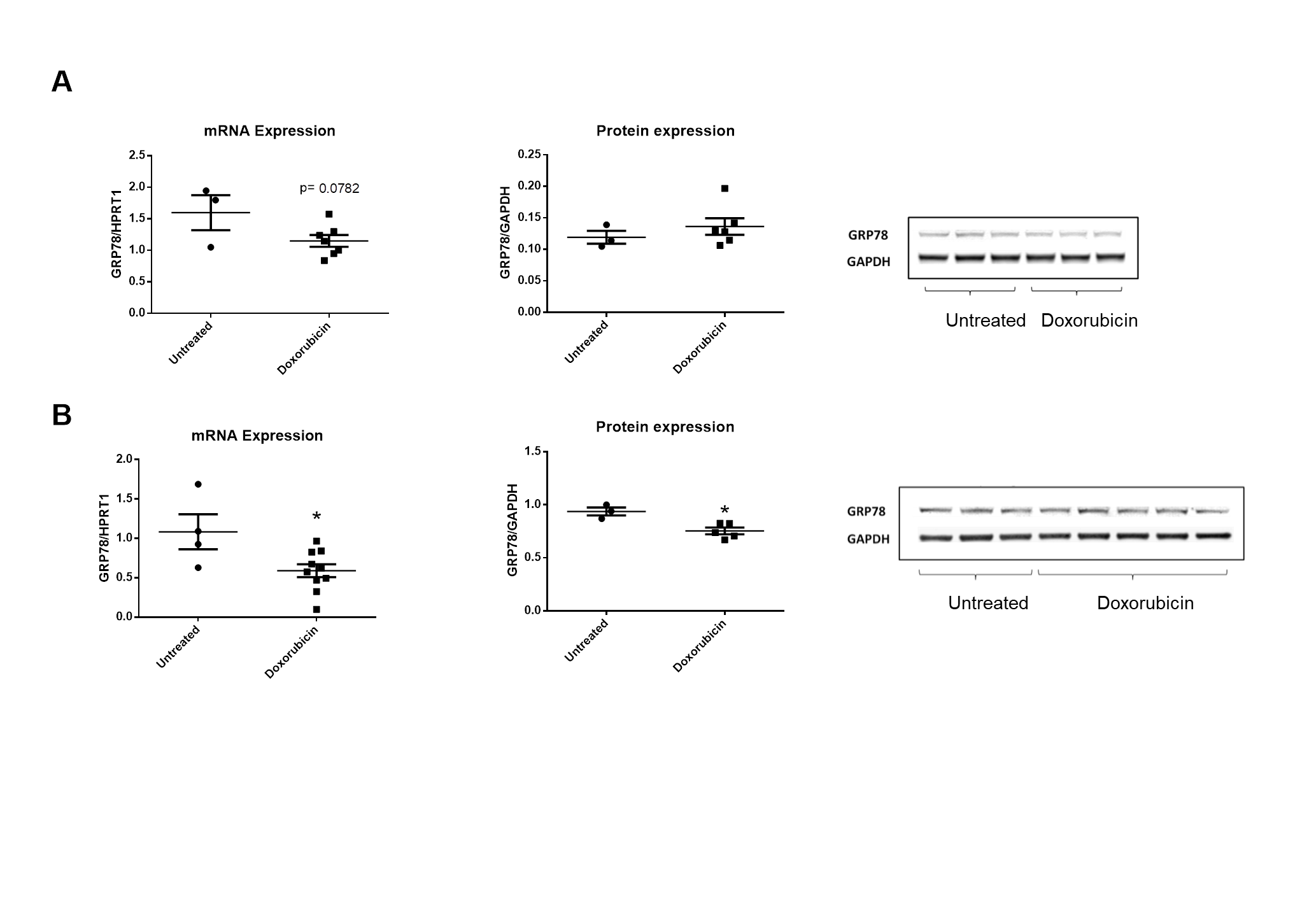

Supplement: S3 Fig — (A) GRP78 mRNA and protein level 24h after i.p.-injection of 20 mg/kg Doxorubicin (Dox) or NaCl (untreated). (B) GRP78 mRNA and protein level 5 days after i.p.-injection of 25 mg/kg Doxorubicin (Dox) or NaCl (untreated). GRP78 levels are significantly decreased. Expression of GRP78 was normalized to HPRT1 mRNA or GAPDH protein. Statistic: unpaired, two-tailed t-Test (* P<0.05). (TIF) [file pone.0215992.s003.TIF]

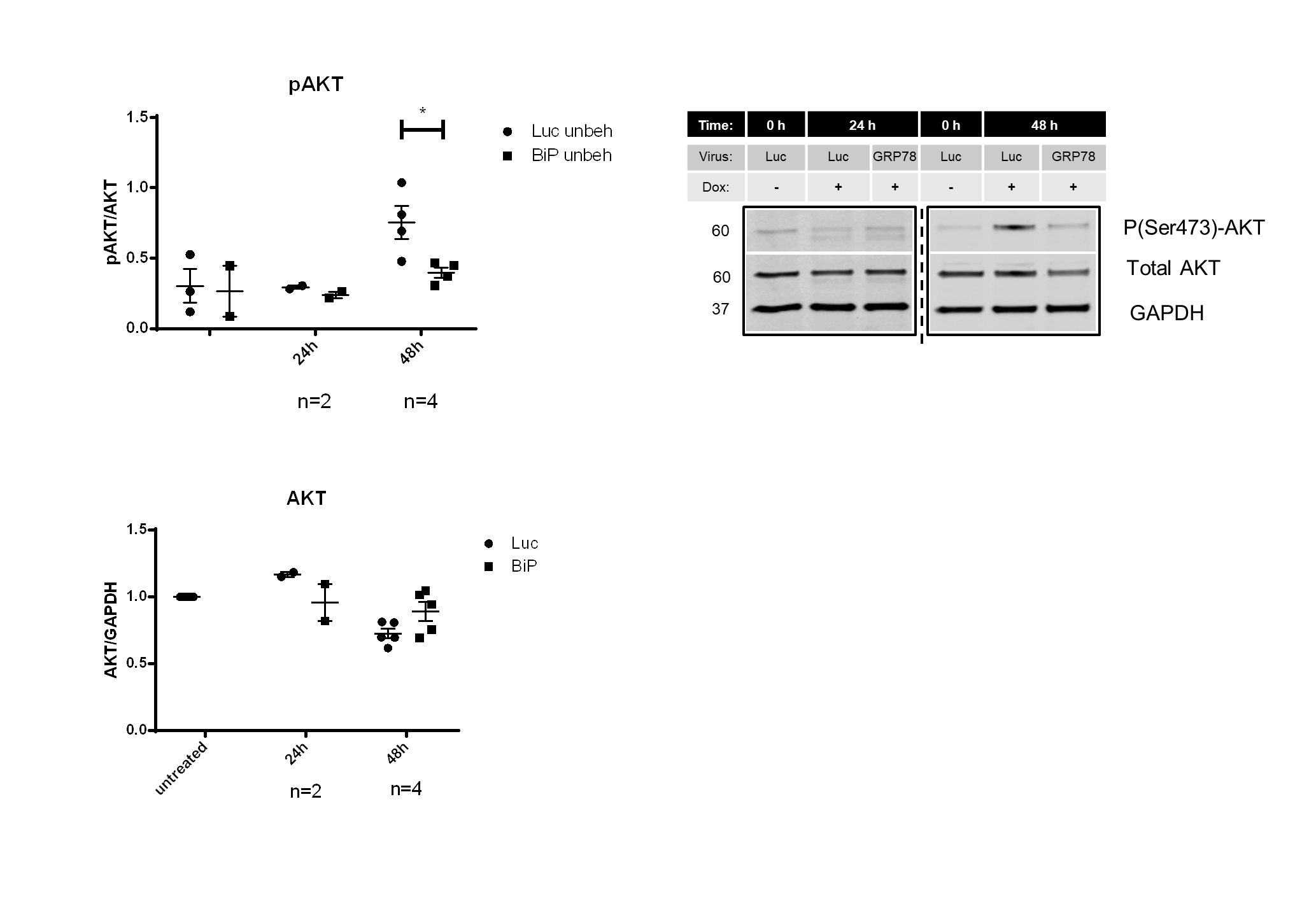

Supplement: S4 Fig — Isolated ventricular cardiomyocytes were transduced with AAV.GRP78 or AAV.Luc and treated with vehicle (untreated) or 1 μM Doxorubicin for 24h and 48h. AKT (Ser473) is significantly phosphorylated after 48h of Dox-treatment, which is diminished by GRP78-overexpression. *P<0.05 for 1-way ANOVA with Bonferroni post hoc testing. (TIF) [file pone.0215992.s004.TIF]

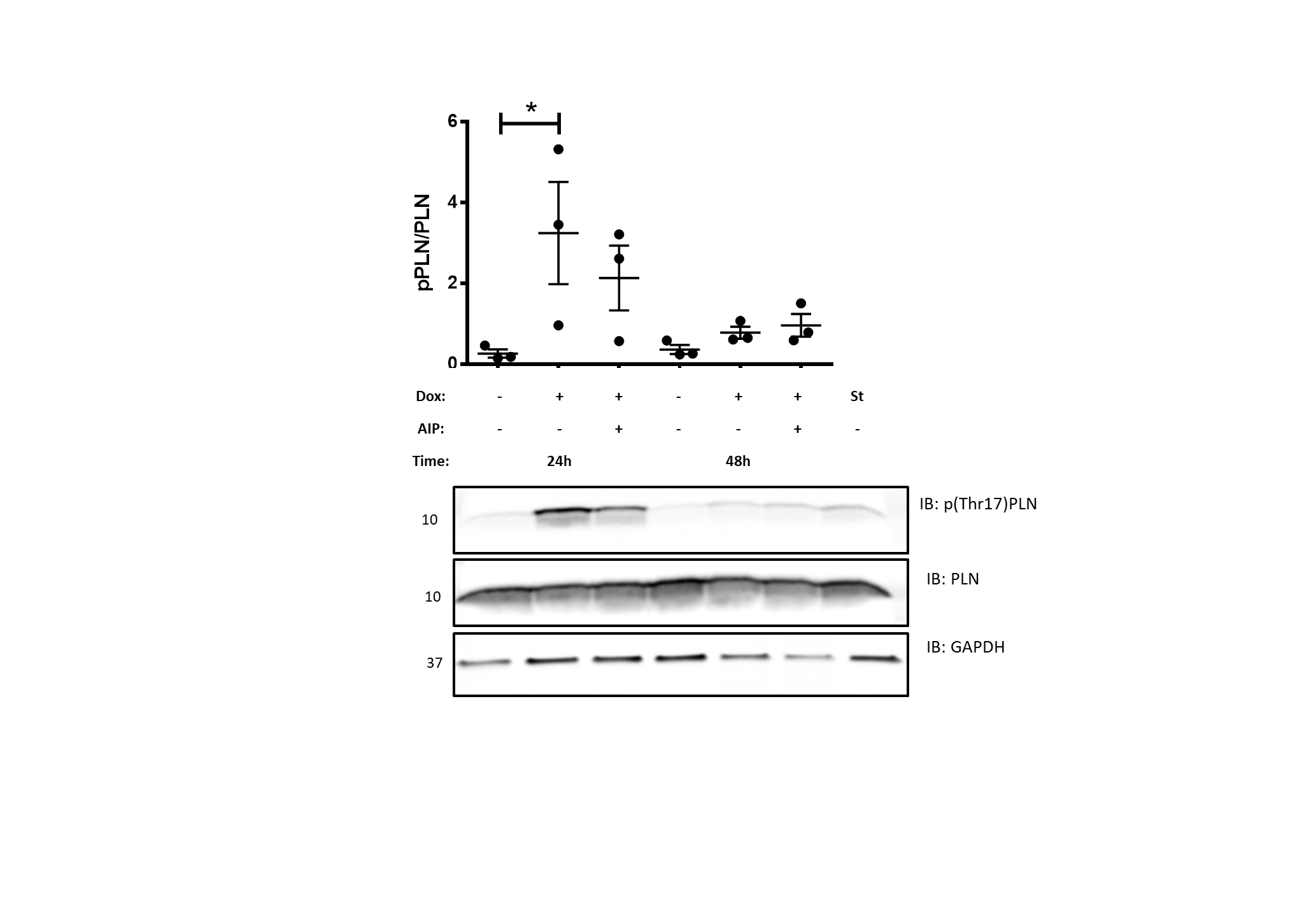

Supplement: S5 Fig — Isolated ventricular cardiomyocytes were treated with vehicle (untreated) or 1 μM Doxorubicin for 24h and 48h. Dox induced significant p(Thr17)-PLN Phosphorylation after 24 h, which was moderately diminished by AIP. *P<0.05 for 1-way ANOVA with Bonferroni post hoc testing. (TIF) [file pone.0215992.s005.TIF]

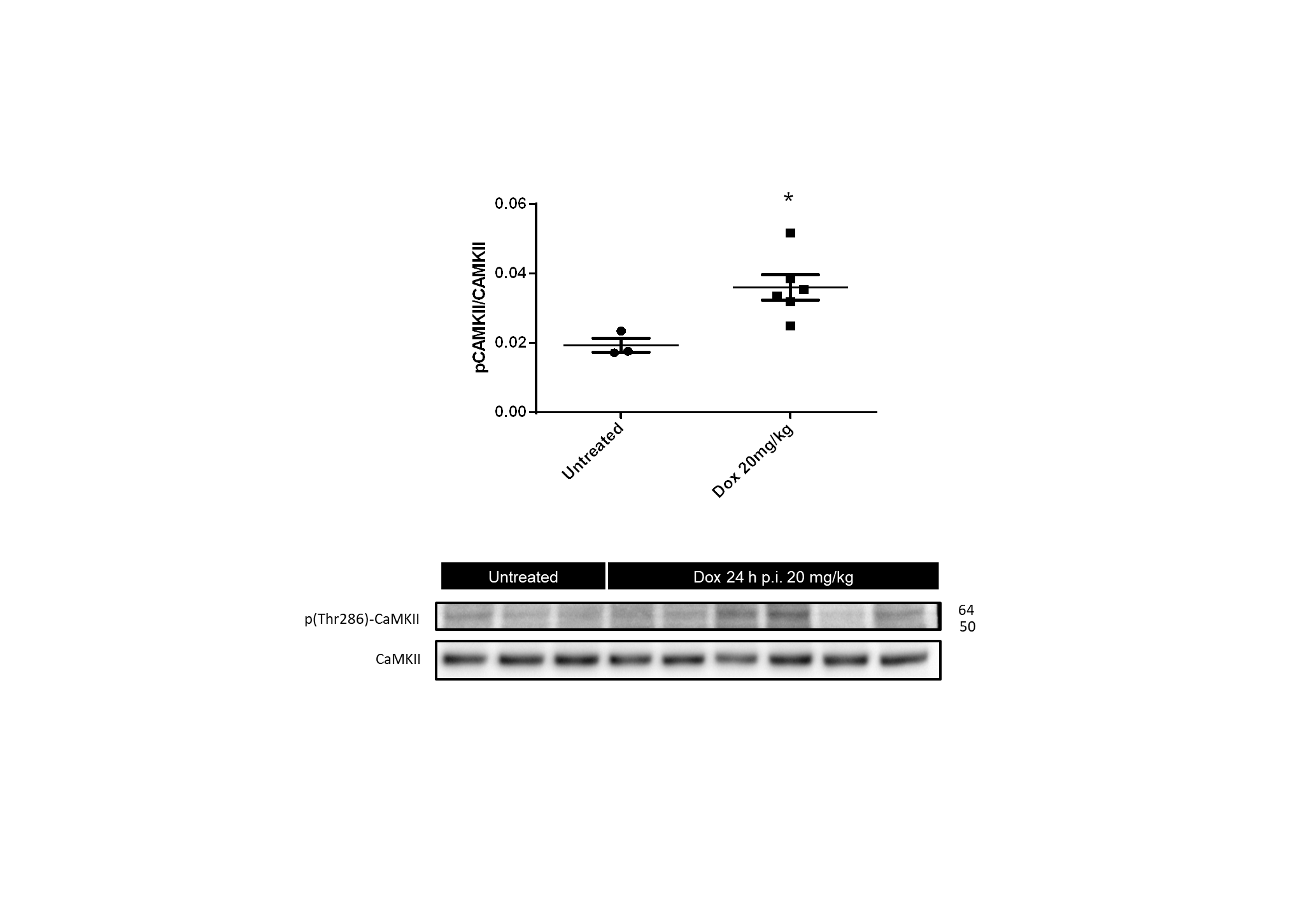

Supplement: S6 Fig — 24h after injecting mice with 20 mg/kg Doxorubicin (Dox) or NaCl (untreated), left ventricular myocardium was used to perform immunoblot (bottom panel). *P<0.05 for Mann-Whitney test. n = 3 (untreated)– 6 (Dox-treated) (TIF) [file pone.0215992.s006.TIF]

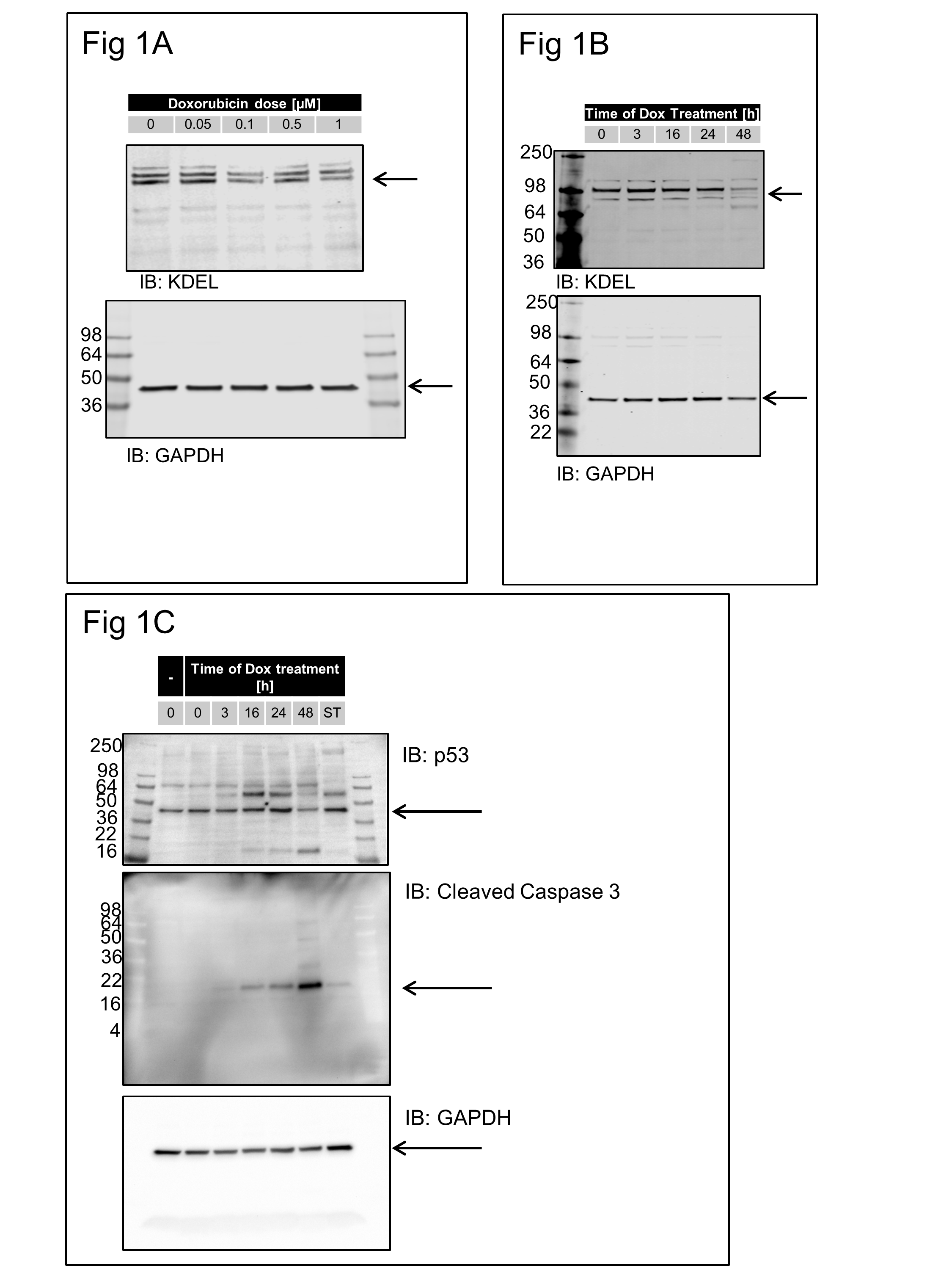

Supplement: S7 Fig — Immunoblots were stained for KDEL and GAPDH (Fig 1A and 1B) or p53, Cleaved Caspase 3 and GAPDH (Fig 1C). St: Sample used for blot-to-blot normalization. (TIF) [file pone.0215992.s007.TIF]

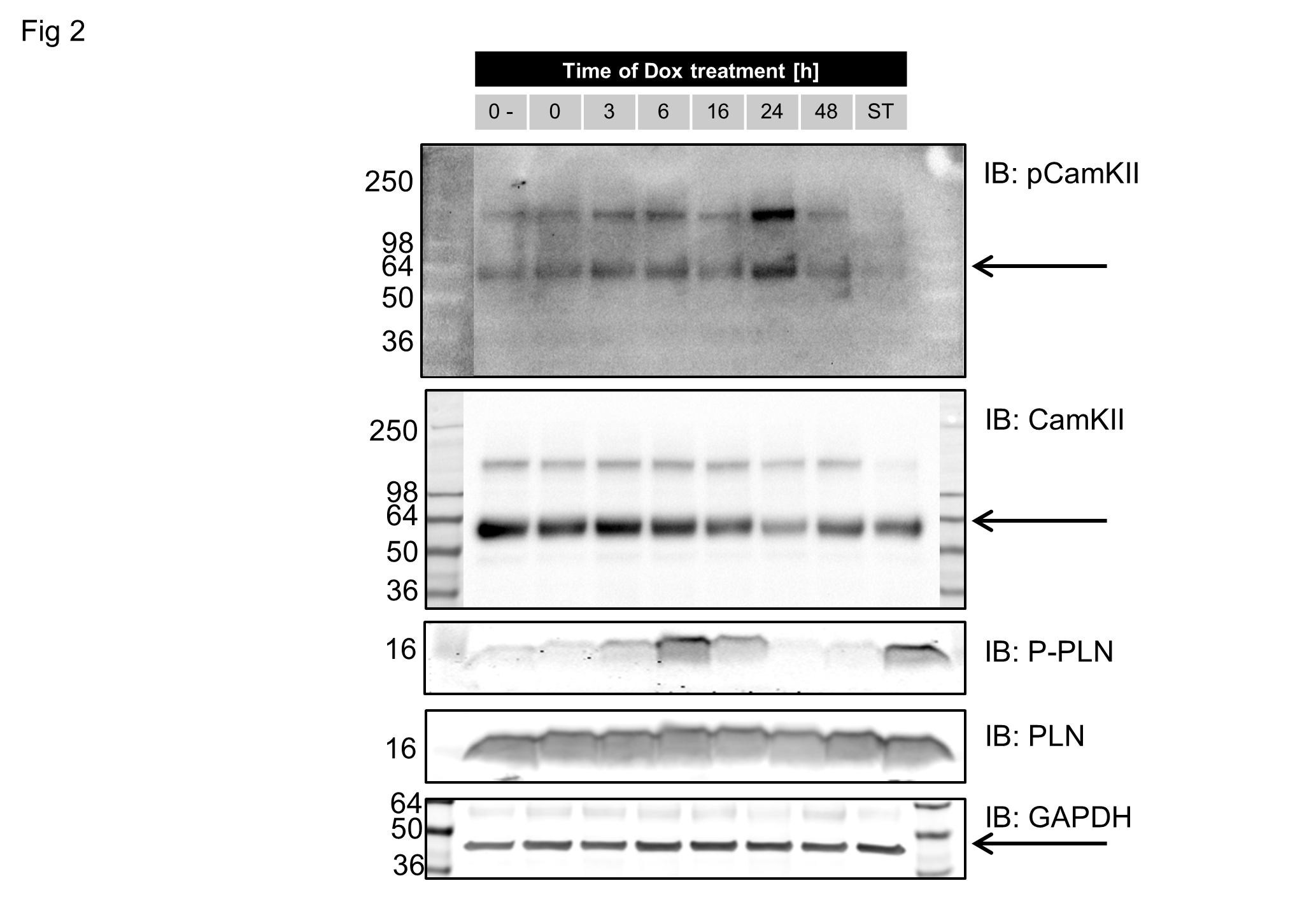

Supplement: S8 Fig — Immunoblot was stained for p-CaMKII (Thr286), CaMKII, pPLN (Thr17), PLN and GAPDH. St: Sample used for blot-to-blot normalization. (TIF) [file pone.0215992.s008.TIF]

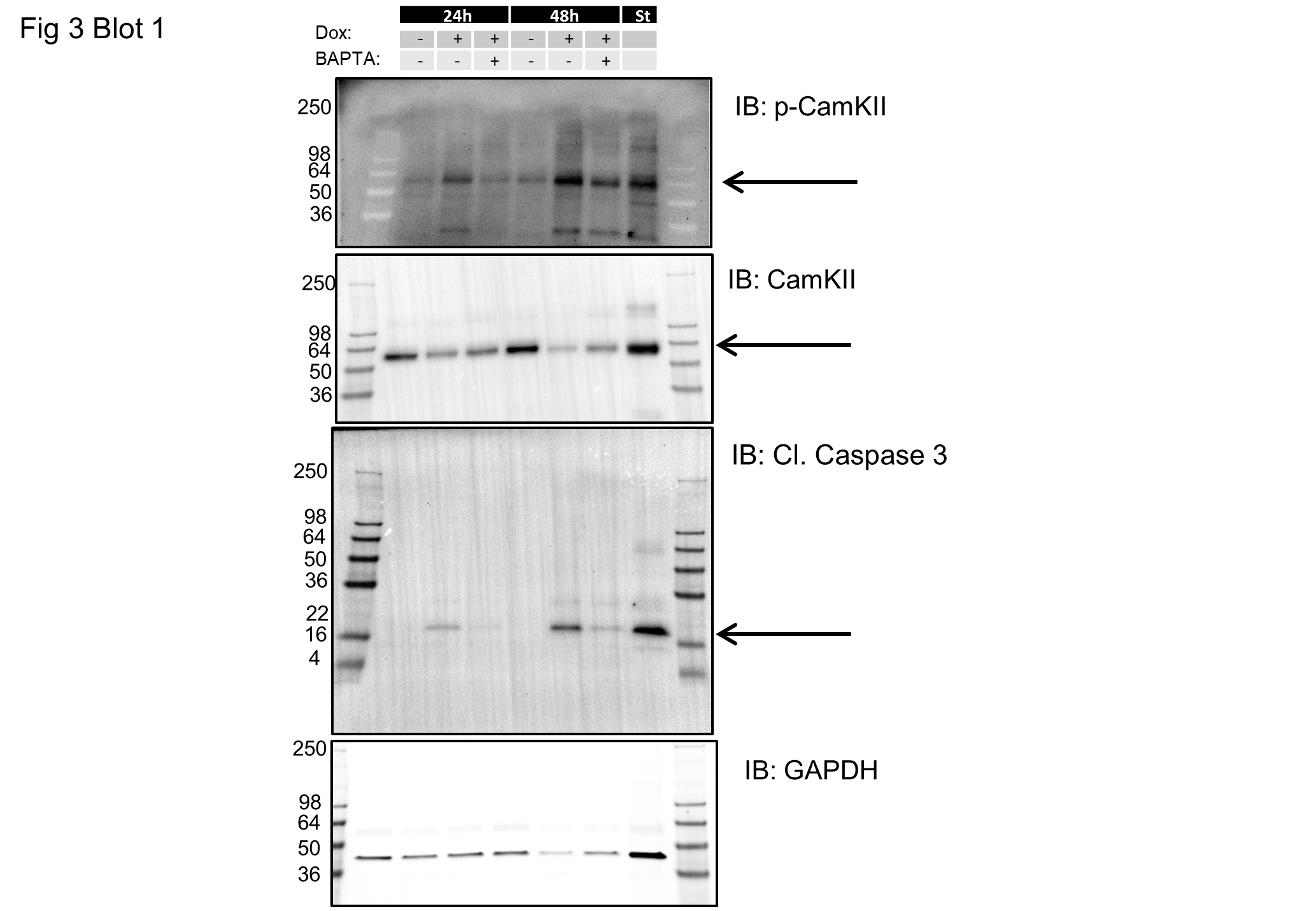

Supplement: S9 Fig — Immunoblot was stained for p-CaMKII (Thr286), CaMKII, Cleaved Caspase 3 and GAPDH. St: Sample used for blot-to-blot normalization. (TIF) [file pone.0215992.s009.TIF]

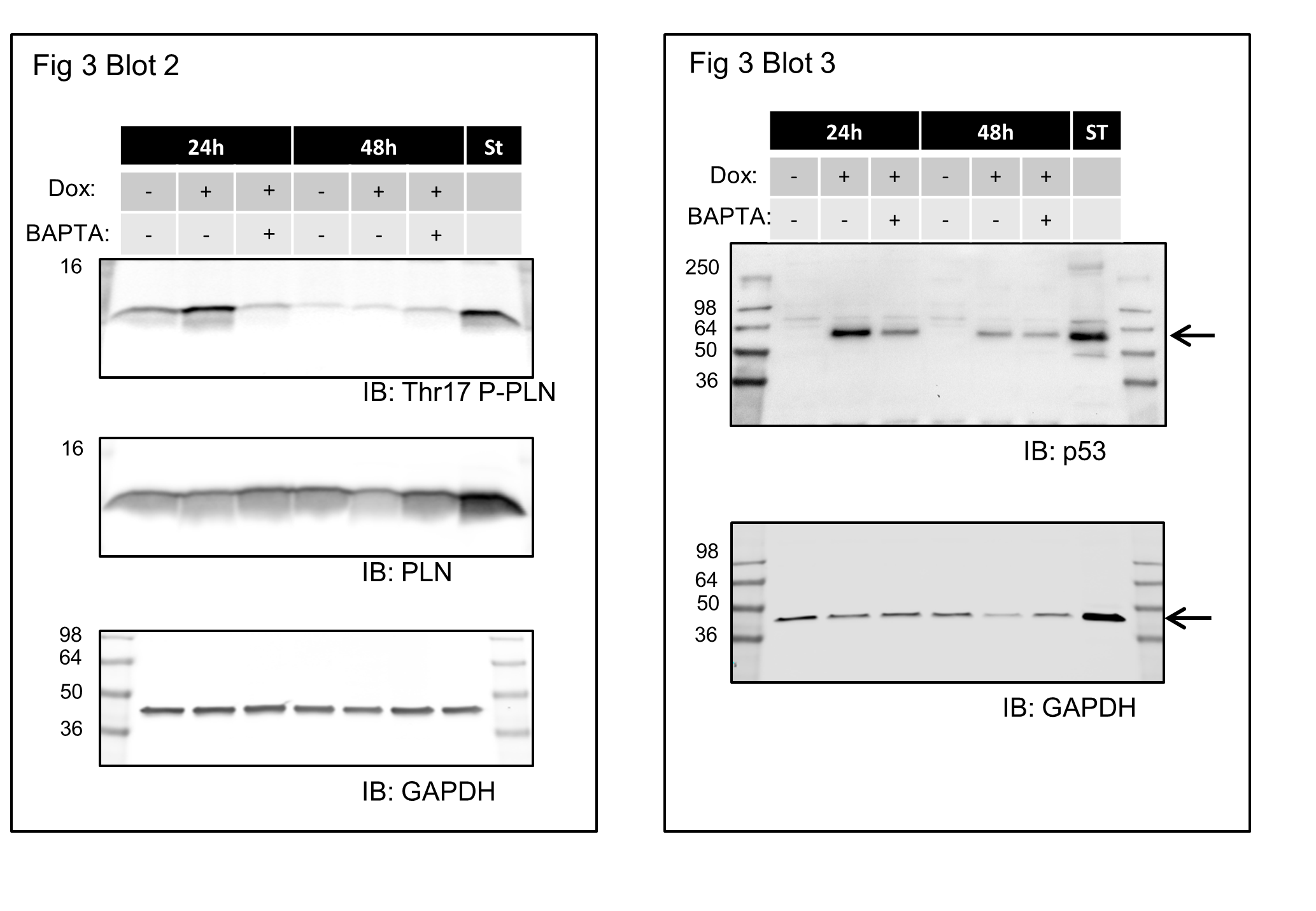

Supplement: S10 Fig — Immunoblots were stained for pPLN (Thr17), PLN and GAPDH (blot 2) or p53 and GAPDH (blot 3). St: Sample used for blot-to-blot normalization. (TIF) [file pone.0215992.s010.TIF]

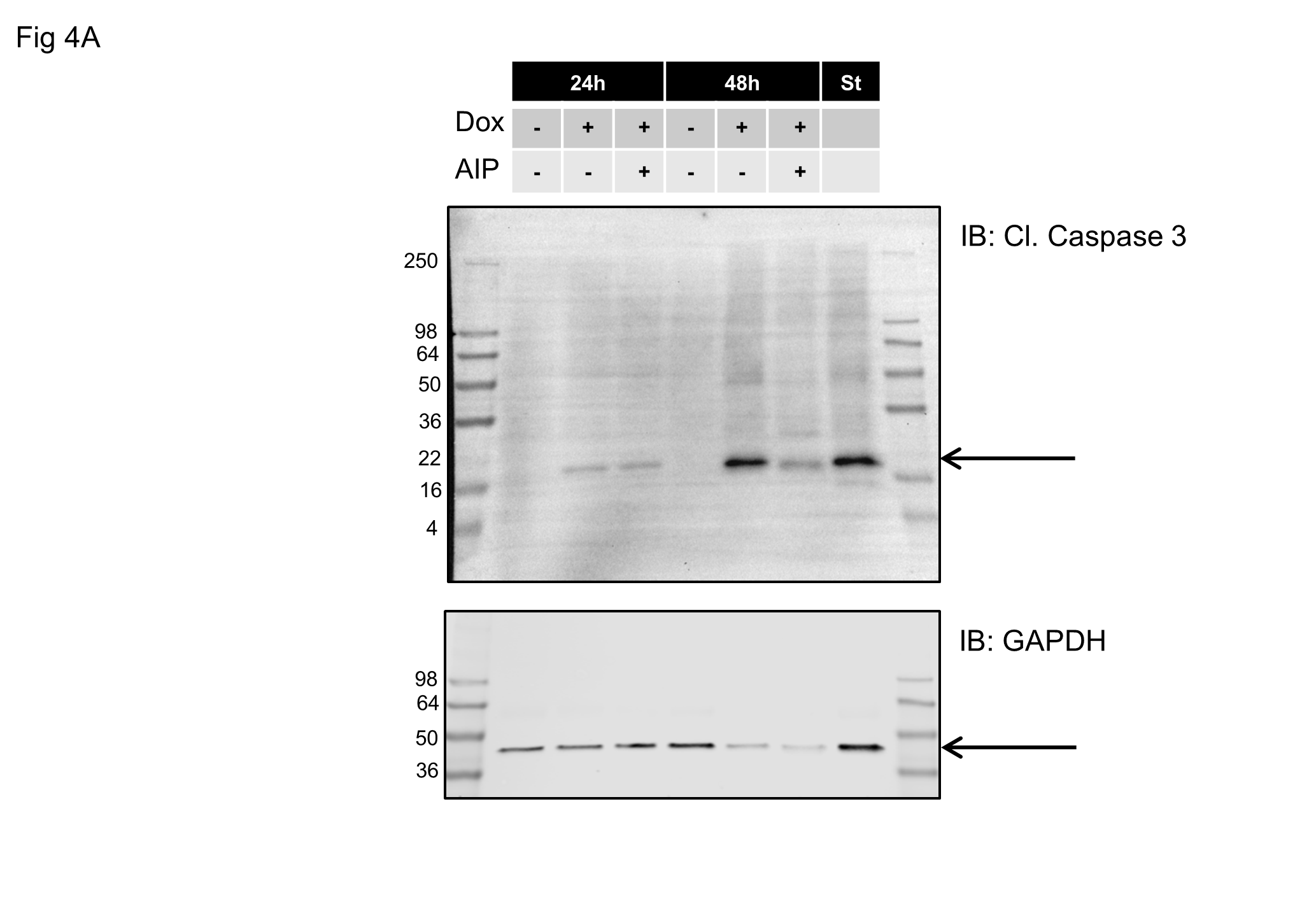

Supplement: S11 Fig — Immunoblot was stained for Cleaved Caspase 3 and GAPDH. St: Sample used for blot-to-blot normalization. (TIF) [file pone.0215992.s011.TIF]

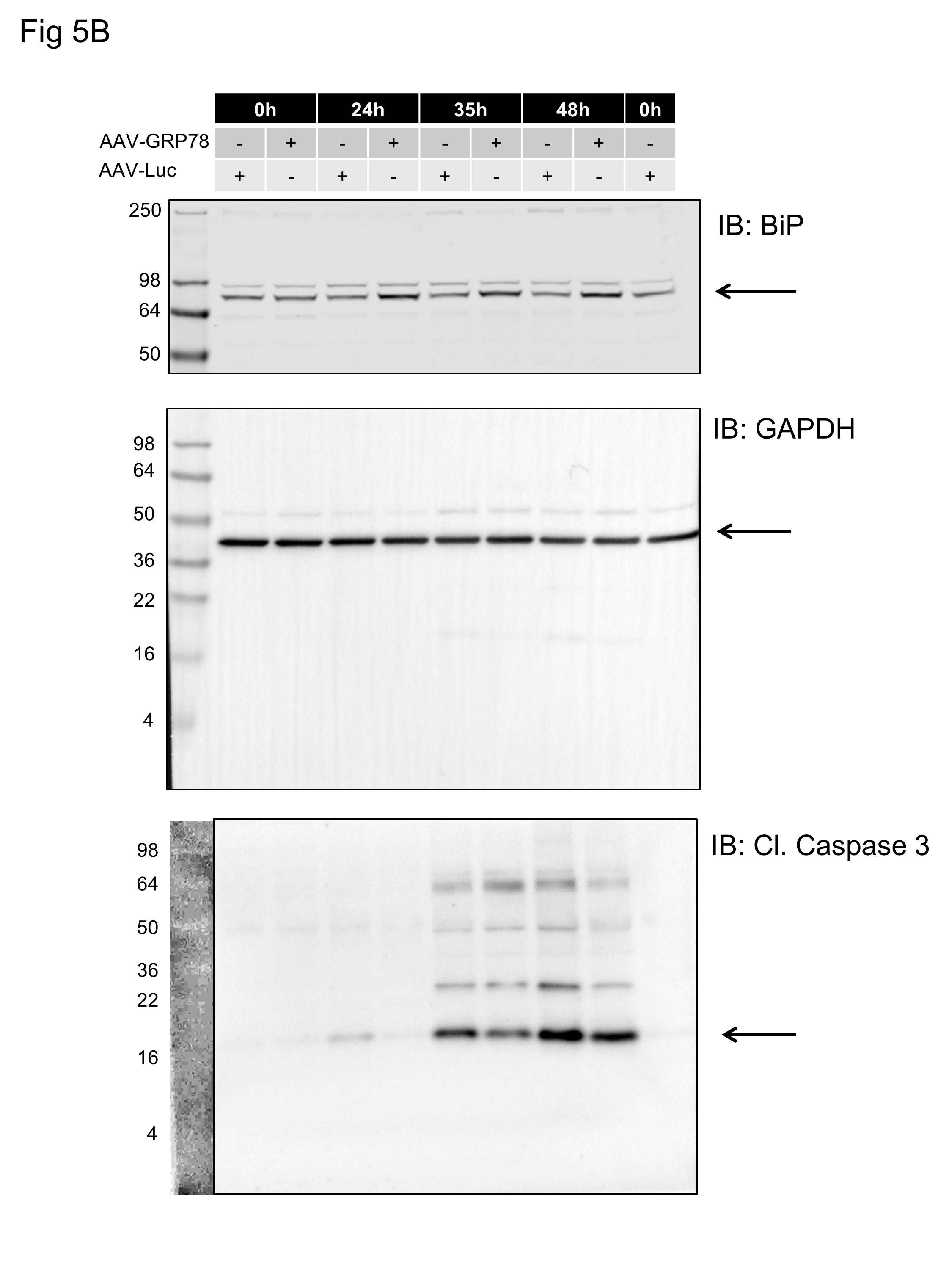

Supplement: S12 Fig — Immunoblot was stained for GRP78/BiP, GAPDH and Cleaved Caspase 3. (TIF) [file pone.0215992.s012.TIF]

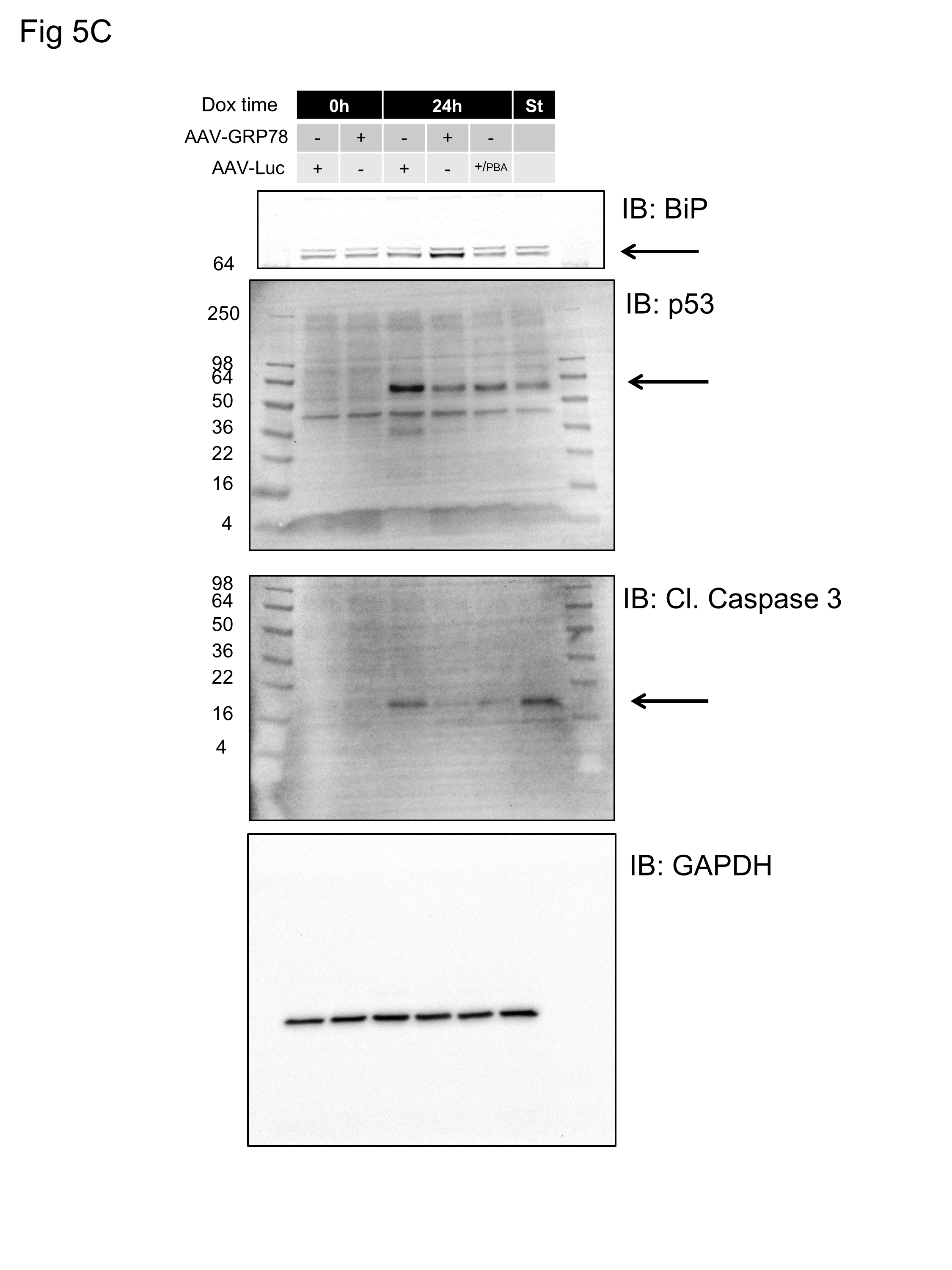

Supplement: S13 Fig — Immunoblot was stained for GRP78/BiP, p53, Cleaved Caspase 3 and GAPDH. St: Sample used for blot-to-blot normalization. (TIF) [file pone.0215992.s013.TIF]

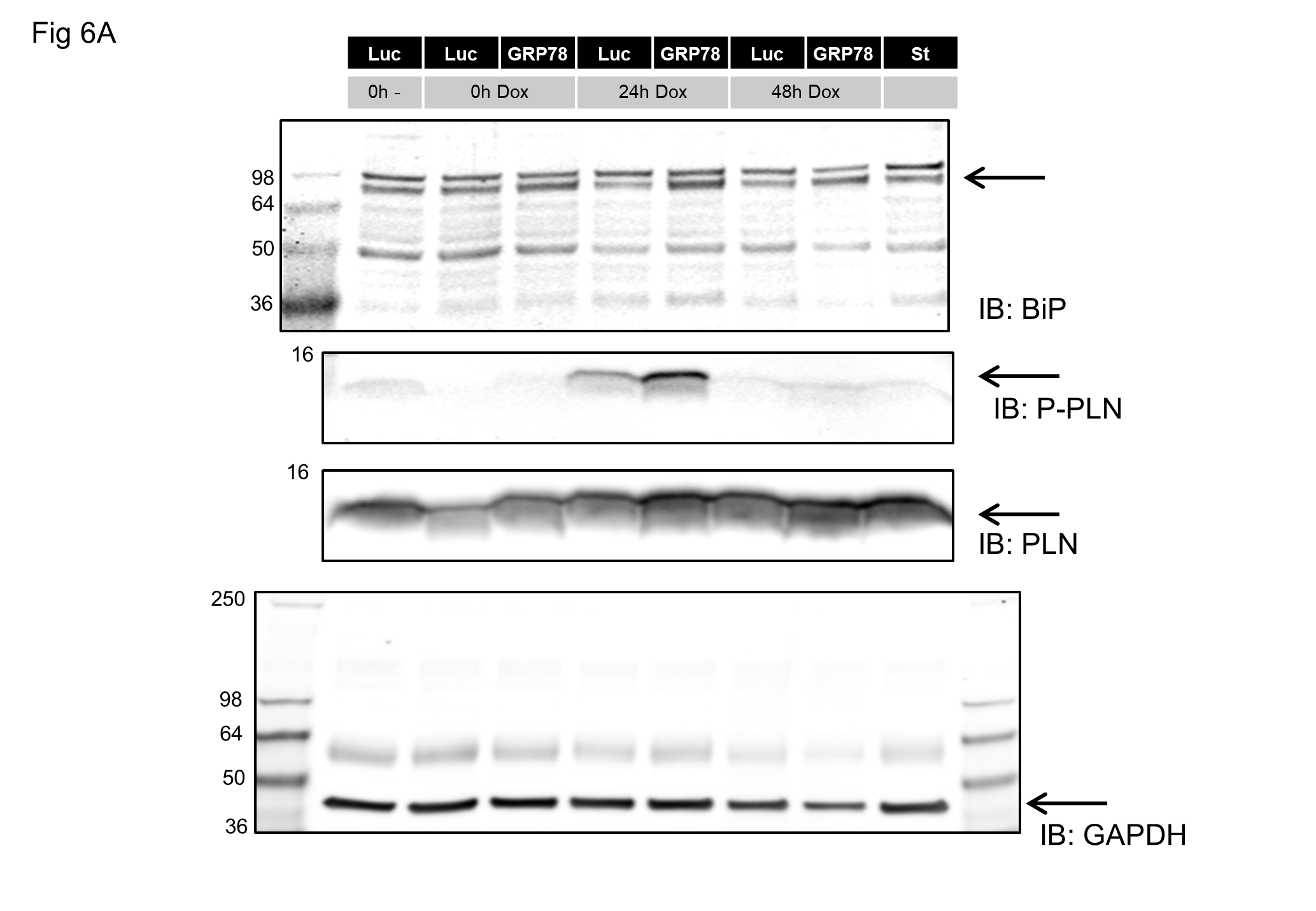

Supplement: S14 Fig — Immunoblot was stained for GRP78/BiP, pPLN (Thr17), PLN and GAPDH. St: Sample used for blot-to-blot normalization. (TIF) [file pone.0215992.s014.TIF]

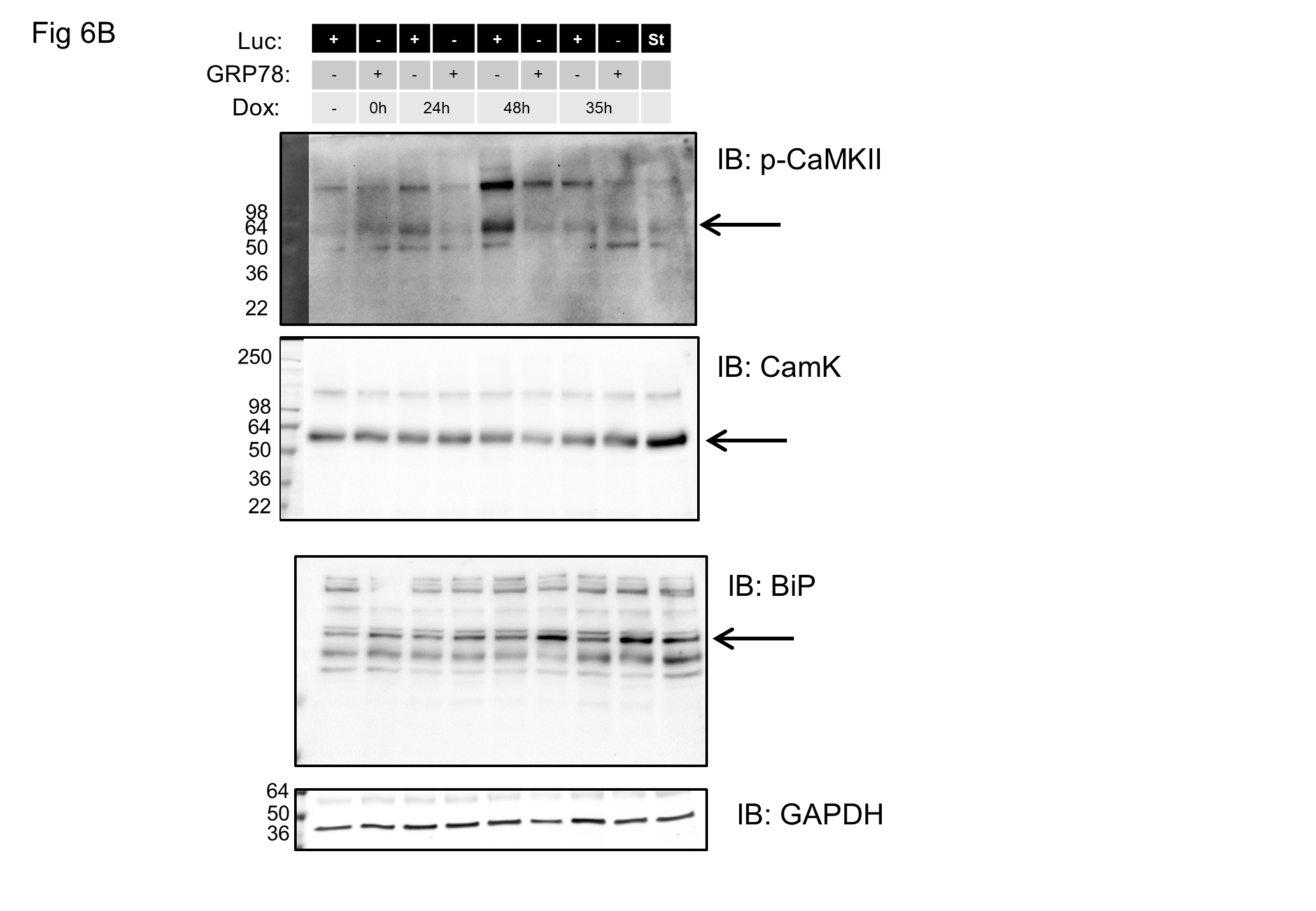

Supplement: S15 Fig — Immunoblot was stained for p-CaMKII (Thr286), CaMKII, GRP78/BiP and GAPDH. St: Sample used for blot-to-blot normalization. (TIF) [file pone.0215992.s015.TIF]

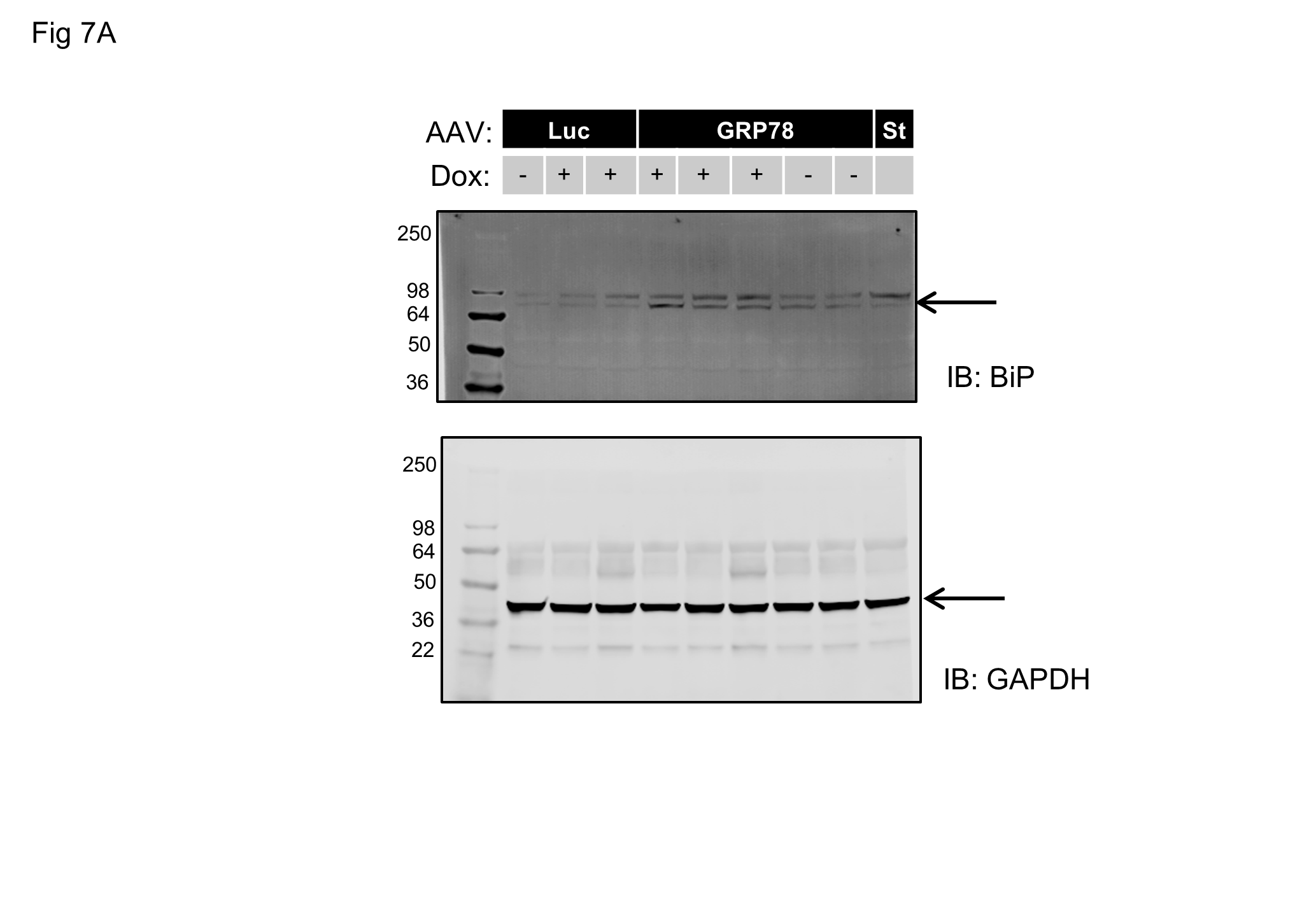

Supplement: S16 Fig — Immunoblot was stained for GRP78/BiP and GAPDH. St: Sample used for blot-to-blot normalization. (TIF) [file pone.0215992.s016.TIF]

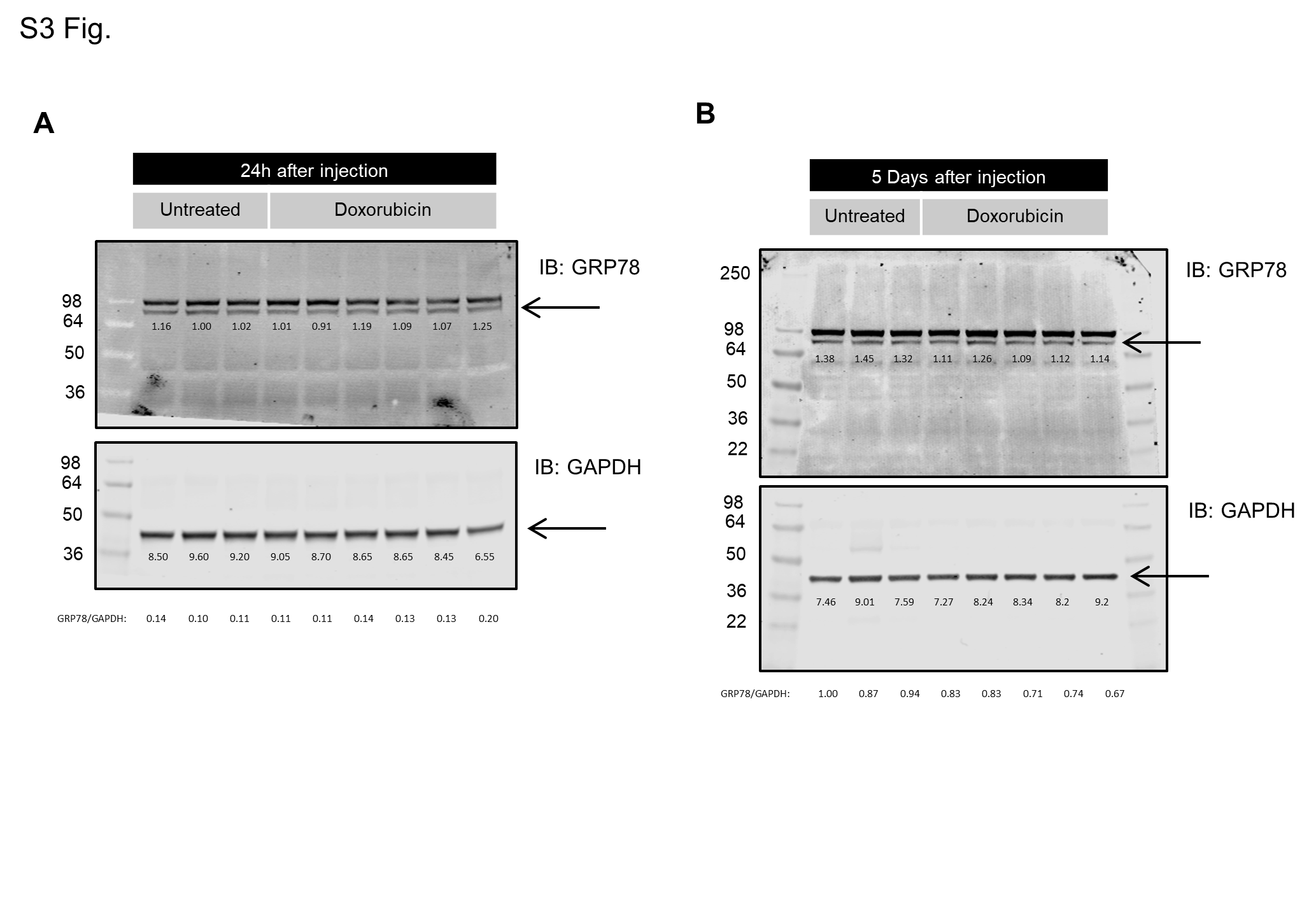

Supplement: S17 Fig — A and B. Immunoblots were stained for GRP78/BiP and GAPDH. For visualization of GRP78 expression, densitometric quantification was added under each band. The ratio of GRP78 to GAPDH is added under the immunoblot. (TIF) [file pone.0215992.s017.tif]

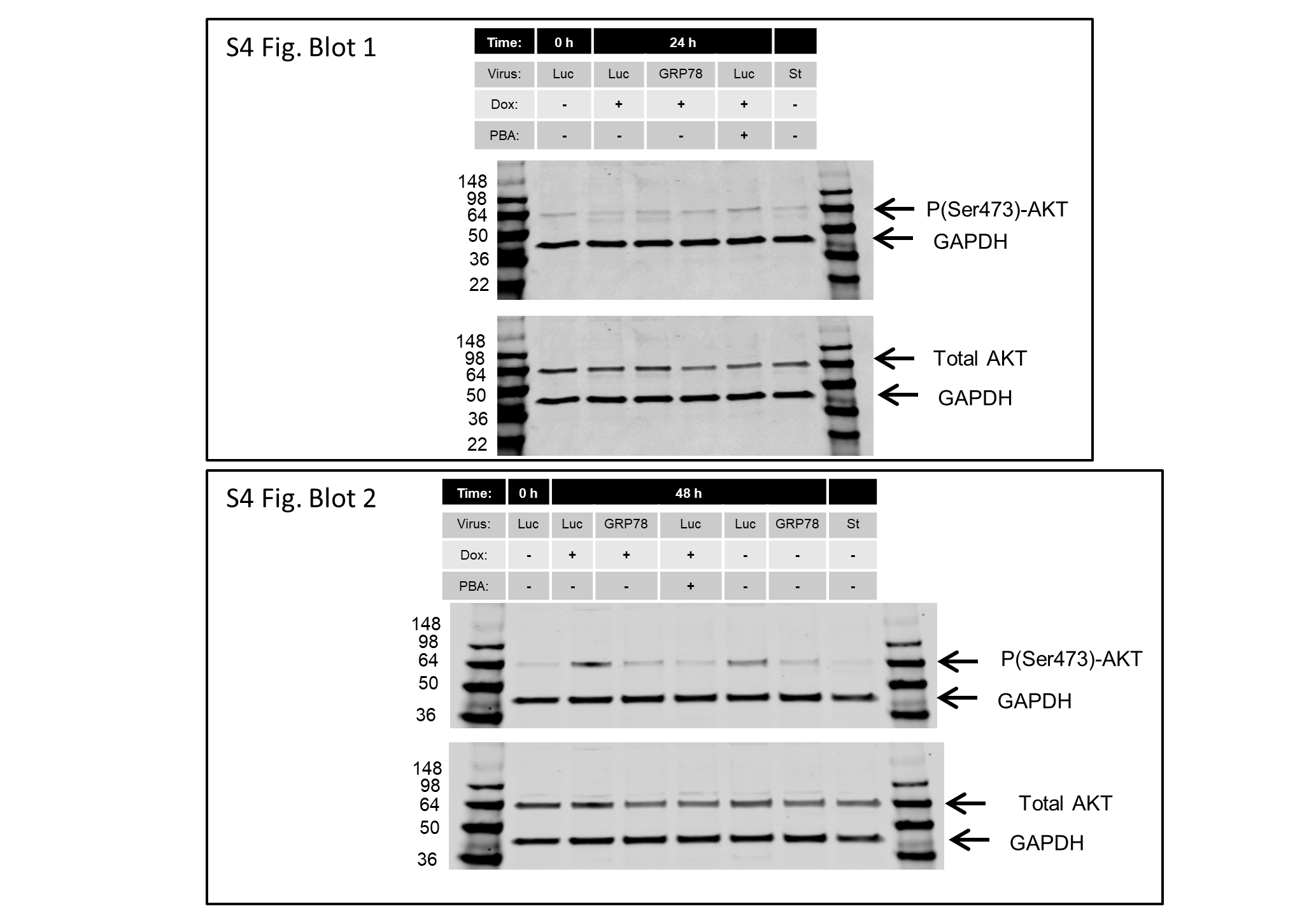

Supplement: S18 Fig — Immunoblots were stained for P-AKT (Ser473), total AKT and GAPDH. St: Sample used for blot-to-blot normalization. (TIF) [file pone.0215992.s018.TIF]

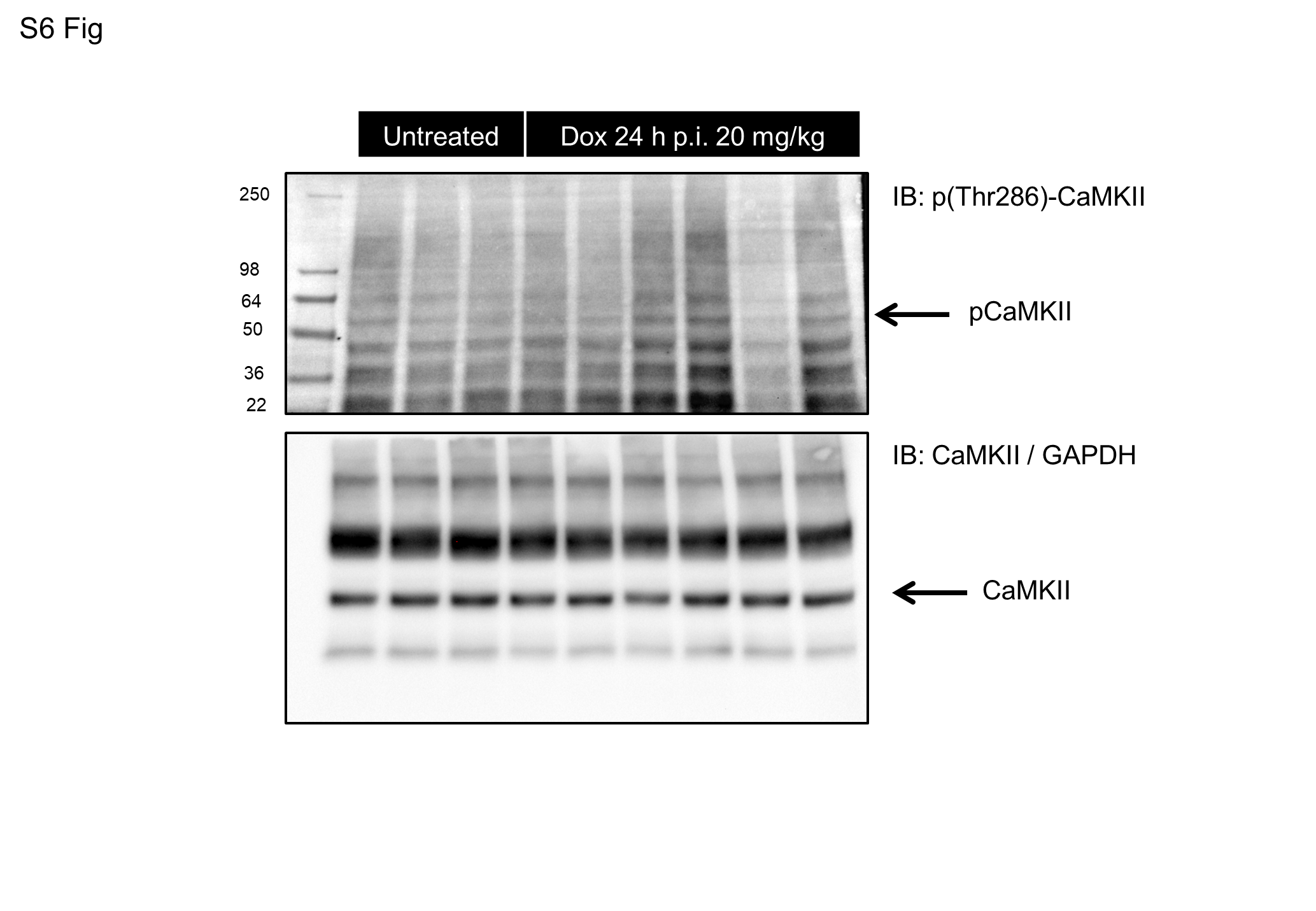

Supplement: S19 Fig — Immunoblot was stained for p-CaMKII (Thr286) and CaMKII. (TIF) [file pone.0215992.s019.TIF]
