# Supplementary material for: CaMKII activation participates in doxorubicin cardiotoxicity and is attenuated by moderate GRP78 overexpression
Source: PLoS One. 2019 Apr 29;14(4):e0215992. doi: 10.1371/journal.pone.0215992 (PMC6488194; doi:10.1371/journal.pone.0215992)
Supplement: S1 File — (DOCX) [file pone.0215992.s020.docx]

# Supplementary methods

## Virus production

High titre adeno-associated virus (AAV) vectors were produced as described before [2]. For production of AAV-GRP78 a vector backbone plasmid containing a GRP78 cDNA (NM_022310.3) under the transcriptional control of a CMV-enhanced myosin light chain (CMV-MLC) 1,8kb promoter was used (pUFCMV-MLC1.8GRP78) [1]. HEK 293T were double transfected with the vector pUFCMV-MLC1.8GRP78 and with pDP9 providing the AAV-9 cap sequence as well as adenoviral helper sequences. Vectors were harvested after 48h, purified by Iodixanol step gradient centrifugation, and quantified using real-time PCR. AAV9-Luciferase control vector was produced with the same method using pUFCMV-MLC1.8-Luc.

## RNA Isolation and Quantitative Real-Time PCR

RNA from adherent cells or 10 – 15 mg tissue was collected using 1ml TriZol reagent (Life Technologies GmbH, Darmstadt, Germany). Tissue was homogenized using ceramic beads in a bead mill for 20 sec. at 5,500 rpm. RNA isolation was done according to the manufacturer’s recommendation. For precipitation of RNA from adherent cells, the water/2-propanol mixture was supplemented with 1.5 µL Polyacryl carrier and incubated at -20°C over night.

The purified RNA was transcribed using iScript cDNA Synthesis Kit (BioRad Laboratories GmbH, München, Germany) according to the manufacturer’s recommendation. 1 µg of RNA was used in 20 µL reaction volume. cDNA was 1:100 diluted and stored at -20°C for subsequent steps.

Real Time PCR was done using SYBR Green Reaction Mix (Bio-Rad) in a 96-well plate setup in a MyiQ Real Time Cycler (BioRad). Following 5’ -> 3’ primers were used: mouse GRP78 (NM_022310.3): fw TGCAGCAGGACATCAAGTTC, rev tacgcctcagcagtctcctt; rat GRP78 (NM_013083.2) fw cctgttgctggactctgtga, rev gaatacaccgacgcaggaat; mouse/rat 18S (NR_003278.3) fw TCAAGAACGAAAGTCGGAGG, rev GGACATCTAAGGGCATCAC; mouse HPRT1 (NM_013556.2) fw GTCCCAGCGTCGTGATTAGC, rev GTGATGGCCTCCCATCTCCT.

## Immunoblot

For protein isolation, tissue samples were homogenized in 100µl/10mg RIPA buffer (150 mM NaCl, 50 mM Tris, 50 mM EDTA, 1 % NP40 (v/v), 0.5 % Desoxycholate,10 mM NaF, 10 mM Na_2_-Pyrophosphate) using ceramic beads in a bead mill for 2 x 30 sec. at 6,000 rpm. Homogenate was incubated on ice for 30 min. For protein isolation from adherent cells, cells were lysed in 50µl RIPA buffer/well and incubated on ice for 30 min. Cell and tissue lysates were centrifuged for 15 min., 18,000 x*g*, 4°C to remove debris. Protein concentration of supernatant was measured using DC Protein Assay Kit (BioRad). Typically 100µg denatured protein were segregated on a 4 – 20 % gradient Tris-Gylcine gel (Thermo Fisher Scientific, Waltham, USA) and transferred to a PVDF membrane (Millipore, Dundee, UK) in a Trans-Blot Semidry blotting chamber (BioRad). After blocking in i-Block blocking reagent (Thermo Scientific, Schwert) for 1h the following antibodies were used diluted in iBlock solution: AKT (rabbit polyclona, CST, Danvers, USA, #9272) 1:1000, GRP78 (rabbit polyclona, CST, #3183) 1:1000, Cleaved Caspase3 (rabbit polyclonal, CST #9661S), CaMKII (mouse monoclonal, BD Biosciences # 611292) 1:1000, pAKT (Ser473, mouse monoclonal, CST, Danvers, USA, #4051) 1:1000, pCaMKII (Thr286, rabbit monoclonal, CST #12716) 1:1000, KDEL (mouse monoclonal, Santa Cruz, #sc-58774) 1:1000, PLN (mouse monoclonal, Thermo Fisher Scientific, # MA3-922) 1:5000, pPLN (Thr17, rabbit polyclonal, Badrilla, Leeds, UK #A010-13AP) 1:5000, GAPDH (mouse monoclonal, Millipore/Merck, Darmstadt, Germany #MAB374) 1:10,000, P53 (rabbit polyclonal, CST #9282) 1:1000. Secondary antibodies were goat-α-rabbit-HRP (SantaCruz #sc-2004), goat-α-mouse-HRP (SantaCruz #sc-2302), Alexa Fluor 680 α-mouse (Thermo Fisher Scientific, Waltham, USA #A-21109), DyLight 800 4X PEG Conjugate α-rabbit (New England Biolabs, Frankfurt (Main), Germany #5151P). For HRP-coupled antibodies, SignalFire ECL Detection Kit was used (CST).

## Isolation of Neonatal Rat Ventricular Cardiomyocytes (NRVCM)

2-3 days old neonatal Wistar rats were decapitated, hearts were collected, washed and then minced in cold ADS buffer *(*120 mmol/l NaCl, 20 mmol/l HEPES, 8 mmol/l NaH_2_PO_4_, 6 mmol/l glucose, 5 mmol/l KCl, 0.8 mmol/l MgSO_4_, pH 7.4). Up to six digestion steps with pancreatin (SIGMA-Aldrich, Munich, Germany, 0.6 mg/ml) and collagenase type II (Worthington, Lakewood, USA, 0.5 mg/ml) in sterile ADS buffer were performed. After each step the supernatant was collected and the pelleted cells stored in FCS (Biochrom/Merck, Berlin, Germany) at 37°C. The combined pellets were resuspended in cold, sterile ADS buffer and cardiomyocytes separated from fibroblasts using a Percoll gradient (GE Healthcare Sciences, Freiburg, Germany) in sterile ADS buffer. The cardiomyocyte phase was collected and washed 2 times in cold, sterile ADS. Cardiomyocytes were resuspended in Medium 199 + 10 % FCS and plated in cell culture plates. Cells were seeded at densities between 0.1 and 0.2 million cells/cm^2^. Growth medium was replaced by Medium 199 + 0.5 % FCS two days after plating, followed by medium renewal every other day.

## Caspase9 and AIF Staining in vitro

1.5x10^4^ NRVCM were seeded on a 96 well glass bottom plate (Greiner Bio-One GmbH, Frickenhausen, Germany) pre-coated with 0.1 mg/mL Collagen-A in PBS (Biochrom GmbH, Berlin, Germany). Two days after seeding, cells were washed with warm PBS and medium was changed to M199 + 0.5 % FCS. At day 4 after seeding, cells were washed and medium was changed to M199 + 0.5 % FCS containing 1 µM Doxorubicin or vehicle. 14 h after beginning of Dox treatment, 5 µM AIP or vehicle was added to the medium. 48 h after Dox-treatment, cells were washed with PBS and fixed with warm 4 % Paraformaldehyde in PBS (Morphisto GmbH, Frankfurt, Germany) for 20 min. Cells were washed 3 times with PBS and permeabilized using 0.2 % Triton-X-100 in PBS for 10 min. After washing twice with PBS, cells were incubated in i-Block blocking reagent for 1 h at room temperature and incubated with primary antibodies (Cleaved Caspase-9, rabbit polyclonal, CST 9507, 1:400 and sarcomeric alpha-Actinin, mouse monoclonal, Sigma A7811, 1:800; AIF (E-1), Santa Cruz sc-13116, 1:400) diluted in i-Block blocking solution overnight at 4°C. Cells were washed 3 times with PBS and incubated in secondary antibodies (ThermoFisher Alexa fluor 488-conjugated goat anti-rabbit (A-11008) and Alexa fluor 594-conjugated goat anti-mouse (A-11032); 1:800 each) for 1 h at room temperature. Cells were incubated in PBS + 300 nM 4',6-Diamidino-2-phenylindole (DAPI; SIGMA Aldrich, Munich, Germany) for 1 min and washed twice with PBS. After the last washing step, PBS was replaced by PBS + 0.1 % Sodium azide. 42 images per well were taken using InCell Analyzer 2200 (GE Healthcare Europe GmbH, Freiburg, Germany). ImageJ Fiji package was used to analyze 20,000 – 50,000 cells per condition.

To quantify cytoplasmic-to-nuclear translocation of AIF, nuclear and cytoplasmic fluorescence signal produced by the AIF-antibody was measured with ImageJ and the ratio between nuclear and cytoplasmic signal intensity was calculated. Here the cytosol was stained with Oregon Orange-488 Phalloidin.

## Sulforhodamine B Assay

Cells were washed with PBS and fixated with ice cold fixation buffer (95% EtOH (v/v)

5% acidic acid (v/v)) over night at -20°C. After washing twice with water, the cells were incubated in the dark in 100µl of SRB solution (0.4% (w/v) sulforhodamine B sodium salt, 1% acidic acid). Following four washing steps with washing solution (1% acidic acid (v/v)) 300µl of Elution buffer (10mM Tris, unbuffered) were added and the cells were incubated on a shaker in the dark for 10 min. 100µl of the supernatant was taken and absorption at 565 nm recorded.

# References:

1. Muller OJ, Leuchs B, Pleger ST, Grimm D, Franz WM, Katus HA, Kleinschmidt JA (2006) Improved cardiac gene transfer by transcriptional and transductional targeting of adeno-associated viral vectors. Cardiovasc Res 70:70-78 doi:10.1016/j.cardiores.2005.12.017

2. Raake PW, Schlegel P, Ksienzyk J, Reinkober J, Barthelmes J, Schinkel S, Pleger S, Mier W, Haberkorn U, Koch WJ, Katus HA, Most P, Muller OJ (2013) AAV6.betaARKct cardiac gene therapy ameliorates cardiac function and normalizes the catecholaminergic axis in a clinically relevant large animal heart failure model. Eur Heart J 34:1437-1447 doi:10.1093/eurheartj/ehr447
